# Mitogenomes from Two Uncommon Haplogroups Mark Late Glacial/Postglacial Expansions from the Near East and Neolithic Dispersals within Europe

**DOI:** 10.1371/journal.pone.0070492

**Published:** 2013-07-31

**Authors:** Anna Olivieri, Maria Pala, Francesca Gandini, Baharak Hooshiar Kashani, Ugo A. Perego, Scott R. Woodward, Viola Grugni, Vincenza Battaglia, Ornella Semino, Alessandro Achilli, Martin B. Richards, Antonio Torroni

**Affiliations:** 1 Dipartimento di Biologia e Biotecnologie “L. Spallanzani”, Università di Pavia, Pavia, Italy; 2 School of Applied Sciences, University of Huddersfield, Queensgate, Huddersfield, United Kingdom; 3 Sorenson Molecular Genealogy Foundation, Salt Lake City, Utah, United States of America; 4 AncestryDNA, Provo, Utah, United States of America; 5 Dipartimento di Chimica, Biologia e Biotecnologie, Università di Perugia, Perugia, Italy; IPATIMUP (Institute of Molecular Pathology and Immunology of the University of Porto), Portugal

## Abstract

The current human mitochondrial (mtDNA) phylogeny does not equally represent all human populations but is biased in favour of representatives originally from north and central Europe. This especially affects the phylogeny of some uncommon West Eurasian haplogroups, including I and W, whose southern European and Near Eastern components are very poorly represented, suggesting that extensive hidden phylogenetic substructure remains to be uncovered. This study expanded and re-analysed the available datasets of I and W complete mtDNA genomes, reaching a comprehensive 419 mitogenomes, and searched for precise correlations between the ages and geographical distributions of their numerous newly identified subclades with events of human dispersal which contributed to the genetic formation of modern Europeans. Our results showed that haplogroups I (within N1a1b) and W originated in the Near East during the Last Glacial Maximum or pre-warming period (the period of gradual warming between the end of the LGM, ∼19 ky ago, and the beginning of the first main warming phase, ∼15 ky ago) and, like the much more common haplogroups J and T, may have been involved in Late Glacial expansions starting from the Near East. Thus our data contribute to a better definition of the Late and postglacial re-peopling of Europe, providing further evidence for the scenario that major population expansions started after the Last Glacial Maximum but before Neolithic times, but also evidencing traces of diffusion events in several I and W subclades dating to the European Neolithic and restricted to Europe.

## Introduction

Evidence from mitochondrial DNA (mtDNA) suggests that a southern dispersal from the Horn of Africa along the Indian Ocean coasts might have brought anatomically modern humans out of Africa ∼60–70 thousand years ago (kya) [1]–[3] although archaeological evidence from Southern Arabian and Indian sites has led some to propose an even earlier exit along the southern route [4]–[6]. ∼15–25 ky later, during the Early Upper Palaeolithic, the first modern Europeans arrived from the Levant [7]–[10]. Archaeologists, linguists, anthropologists and, more recently, geneticists have long debated the role of the major colonization and diffusion events in shaping the structure of modern Europeans.

A fundamental question has concerned the relative amount of genetic input into modern Europeans from Palaeolithic *versus* Neolithic waves of settlement. Palaeolithic events include both the first entry to the continent and the re-settlement from southern refugia after the Last Glacial Maximum (LGM), starting from ∼19 kya, while Neolithic phases primarily coincide with the spread of agriculture and pastoralism that began in the Near East ∼10 kya and progressively reached the Balkans, Central Europe, the West Mediterranean, and the north, argued to have been accompanied by substantial increases in population size [11]. As for the relative extent of the genetic traces left by these key events, the debate has been inconclusive. Early analyses based on “classical” genetic markers were interpreted as supporting a Neolithic wave of advance that played a major role in shaping the genetic variability of Europeans [12], [13] with Mesolithic foragers contributing minimally to the present-day genetic background. Subsequently, the analysis of mitochondrial DNA variation based on the phylogeographic analysis of the mtDNA control-region sequence and coding-region RFLP markers [14]–[16] turned the tide, pointing to a more significant contribution from the indigenous hunter-gatherers estimated to at least ∼80%. This suggested that only small groups of Neolithic people settled Europe and a wide-scale adoption of agricultural technology by indigenous Mesolithic/Palaeolithic populations occurred [3].

With the advent of complete mtDNA genome sequencing, clear Palaeolithic and Mesolithic signals in Europe have been retrieved from various mtDNA clades tracing Late Glacial and postglacial expansions of populations from European refuge areas from ∼18 kya, albeit with the majority clustering in the postglacial ∼11.5 kya [1], [3], [17]–[21]. More recently, Pala et al. [22] have further shown that the widespread West Eurasian haplogroups J and T share a common origin in the Near East and expanded at the end of the Last Glacial Maximum from a Near Eastern glacial refuge. Lineages within these haplogroups had previously been identified as potentially accompanying the spread of the Neolithic, but the improved resolution of complete mtDNA genomes showed that the initial move to Europe had been much earlier. However, despite some criticisms [23], the picture of the peopling of Europe with limited, but not insignificant, Neolithic immigration into a mainly Palaeolithic/Mesolithic genetic background, was supported by the analysis of the other uniparental genetic system, the non-recombining, male-specific portion of the Y chromosome [24], which not only confirmed the presence of Mediterranean/Southern European refuges [25], but also traced the genetic legacy of other glacial refuge areas, such as the Balkans and the Periglacial areas of the Ukrainian plains [26], [27].

Ten years after the release of the whole human genome reference sequence [28], [29], previously inconceivable progress has been made in the field of population genetics, with the double aim of both correlating population structure with genetic bases of common diseases and/or drug response and understanding past history and migrations of our species [30]. The future of population genetics will be likely dominated by personal genomics and sequencing of complete genomes at population levels [31], in the meantime genome-wide SNP arrays have contributed to outline continental and population genetic maps. For example, a close correlation between genes and geography (albeit for a tiny fraction of the variation) has been detected within the European continent [32], [33]. However, some of the major questions concerning the peopling of the world remain unanswered, mainly because of the lack of reliable chronologies on detected genetic admixtures and structures [34]. In this respect, mtDNA remains at the forefront of the field, due both to a well-developed molecular clock [21] and the recent accumulation of ancient DNA evidence.

### The Ancient DNA Perspective

A key role in distinguishing the relative amount of Mesolithic *versus* Neolithic genetic traces retained within modern human populations is played by ancient DNA studies, despite problems such as contamination (with consequent misleading selection of rare variants), and small sample sizes contributing to potentially biased views of the ancient gene pool [35]. The earliest farming culture in Central Europe, the Linear Pottery Culture (LBK, from *Linienbandkeramik*), has been precisely dated to ∼7 kya, thanks to recently recalibrated radiocarbon dating, and it also represents the best genetically characterised trace of the Neolithic advent in Europe. A first analysis of 24 Neolithic skeletons from Central Europe, dating back to the LBK period, found that 25% of mtDNAs belonging to haplogroup N1a, were detectable at a 150 times lower frequency (0.2%) in modern Europeans [36]. These controversial [37]–[39] results initially suggested that cultural diffusion was the major mechanism of spread for Neolithic technologies; and, at least for the maternal lineage, the first central European farmers did not significantly contribute to the genetic pool of Europeans, who appear by default to have been of mainly Mesolithic origin. On the other hand, a demic diffusion model was the interpretation of mtDNA sequences obtained from a Spanish Neolithic site dating to 5,500 years BP [40]. Despite the limited sample size (N = 11), the haplogroup composition of the Neolithic population suggested genetic continuity between ancient and modern Iberians. This raised the possibility of heterogeneous patterns of Neolithic dispersal between Central and Southern Europe.

Bramanti and co-workers claimed to have resolved the issue [41] by comparing directly (and for the first time) ancient DNA from skeletons of pre-Neolithic European hunter-gatherers and early farmers (20 and 25 specimens, respectively). The mtDNAs fell into two profoundly distinct groups, thus suggesting genetic discontinuity between Palaeolithic/Mesolithic (mainly carrying haplogroup U) and Neolithic groups, and also between pre-Neolithic and modern populations. More recently, the LBK population sample was increased (to N = 42) and comparisons with modern mtDNA variation suggested a demic (not only cultural) diffusion model of genetic input from the Near East/Anatolia into Central Europe with the early Neolithic [42], as claimed by some anthropologists [43], [44]. However, despite some signs of Neolithic ancestry among modern Europeans, distinct patterns of haplogroup frequency distribution between ancient and modern samples suggest that further major demographic events shaped the genetic landscape of Europe [42]. In conclusion, to quote Rowley-Conwy, “the picture is more complex and, thus, more interesting than these simple scenarios suggest”, with many, maybe as yet undetected, local migratory pulses similar to leapfrog migrations [45].

The concept that the rise of farming and expansion were not uniform processes across Europe has been further corroborated by ancient nuclear genomics. The recent analysis of 5-ky-old skeletal remains from Scandinavia revealed a close genetic link between a Neolithic individual and modern Mediterranean Europeans, while Scandinavian hunter-gatherers clustered with modern northern Europeans (Finns in particular) [46]. Thus, while the classical scenarios envisioned expansions (cultural *versus* genetic) from the Near East towards Europe, forthcoming ancient genomic data are now deciphering internal routes spanning the Neolithic within Europe.

### A Methodological Revolution

Continuous progress in automatic high-throughput sequencing technologies has contributed to a new methodological revolution within mtDNA population studies, allowing entry into public web databases with a large volume of complete mitogenome data, including a recent augmentation with 8,216 modern mtDNA genomes [47]. This revolution has made available to the scientific community a constantly increasing amount of molecular data, raising the level of resolution of human mtDNA phylogeny in terms of haplogroup definition to unprecedented levels [47], [48] (PhyloTree Build 15). This newly available dataset, when constantly enlarged and analysed with emerging and/or well-established phylogenetic/phylogeographic methods, constitutes an extremely informative source of inferences on human evolution and population relationships. The ideal phylogeny will span all worldwide modern human populations, thus including representatives of virtually all extant mtDNA haplotypes. The final aim is to reconstruct and trace, step by step, the journey that our ancestors took to across the world, providing answers to pivotal questions that still remain unsolved. Here are some examples: the contribution of Palaeolithic glacial refugia to Late Glacial and postglacial re-peopling of Europe seems clear, but are there also some traces of European Neolithic migration events clearly marked by mtDNA lineages? Could they clarify major events of the peopling of Europe? In this context, how can we detect the genetic contribution of present-day variation of the numerous demographic events that have taken place in post-Neolithic Europe? Is there continuity or discontinuity between modern and both Palaeolithic and Neolithic ancient DNAs? When discontinuity is detected, is this due to poor dataset resolution and could the above-mentioned “ideal” worldwide phylogeny resolve this question? Some mtDNA haplogroups, sharing peculiar characteristics, are suitable candidates to potentially answer these and other fundamental questions, but often in the past, the level of resolution has been inadequate.

The work presented here focuses on the phylogenetic and phylogeographic analysis of two West Eurasian haplogroups, namely I (within haplogroup N1a1b) and W, which are widely distributed over the entire European continent, the Near East and West Asia, but at low frequencies. Haplogroups I and W split directly from N1 and N2, respectively, thus they are both one step from the root of haplogroup N, the most ancient non-African (or better out-of-Africa) lineage that entered first Southwest Asia (∼60 kya) and then Europe (∼45 kya). A recently published phylogenetic analysis of haplogroups N1 and N2 (including representatives of both haplogroups I and W), as well as haplogroup X, suggested that these clades did indeed represent ancient relicts of the first human dispersals out of Africa along the southern coastal route, localizing their putative origins in the Arabian peninsula [49].

The current human mtDNA phylogeny does not equally represent all human populations but is biased in favour of representatives originally from north and central Europe [47]. This affects the phylogeny of many West Eurasian haplogroups, including I and W, whose southern European and Near Eastern components are poorly represented. The aims of this work were to (i) expand and re-analyse the available datasets of I and W complete mtDNA genomes, reaching a comprehensive 419 mitogenomes (192 I, with the addition of four samples belonging to the poorly represented sister clade N1a1b1– former N1e, and 223 W), by adding 58 new complete sequences, mainly from southern European and Near Eastern individuals, and (ii) accurately define the phylogenetic relationships within subclades of limited geographic distribution and low frequencies, searching for precise correlations between mtDNA haplotypes/clades and events of human dispersal. Our results showed that haplogroups N1a1b1, I and W most probably originated in the Near East during the Last Glacial Maximum or pre-warming period (the period of gradual warming between the end of the LGM, ∼19 kya, and the beginning of the first main warming phase, ∼15 kya) and, like J and T, may have been involved in Late Glacial expansions starting from the Near East. Thus these data contribute to better defining the Late and postglacial re-peopling of Europe, providing further evidence for the scenario that major population expansions started after the Last Glacial Maximum but before Neolithic times.

## Materials and Methods

### Sample Selection and Analysis of mtDNA Sequence Variation

We searched our database of control-region sequences (and relative haplogroup classification based on coding-region markers) from almost 10,000 available subjects of various geographic origins (Africa, East and South Asia, the Near East, Caucasus and Europe) and selected 58 mtDNAs (31 W, 26 I and 1 N1a1b1) for complete mtDNA sequencing. Both control-region variation and geographic/ethnic origin were used as selection criteria, particularly focusing on samples from Mediterranean Europe and the Near East (following the same definition of this term as in [22]). For all subjects involved, appropriate written informed consent was obtained, and the study was approved by the Ethics Committee for Clinical Experimentation at the University of Pavia, Board minutes from October 5th, 2010. These 58 mitogenomes were analysed together with 225 (89 I, 3 N1a1b1 and 133 W) previously available from published data and public databases (*i.e.* NCBI and 1000 Genomes Project) and 136 (77 I and 59 W) made available by recent phylogenetic updates from Behar et al. [47] for a total of 419 (196 belonging to N1a1b and 223 belonging to W) mitogenomes used to build the corresponding phylogenies. Geographic and ethnic affiliations of the 419 mitogenomes are listed in Table S1 and Table S2 in File S1, together with their GenBank or 1000 Genomes Project accession numbers.

We amplified and sequenced mitogenomes following well-established protocols, as reported elsewhere [50], and aligned, assembled, and compared them using Sequencher 5.0 (Gene Codes Corporation), relative to both the newly proposed Revised Sapiens Reference Sequence (RSRS) [47] and rCRS [51]. We performed phylogenetic construction using a maximum parsimony approach with the aid of the mtPhyl software (http://eltsov.org/mtphyl.aspx), correcting the trees by hand with reference to PhyloTree. We assigned haplogroup labels following the nomenclature proposed by the PhyloTree database (at http://www.phylotree.org/) [48]. We obtained maximum likelihood (ML) molecular divergences with the same methodological approach reported in [22] and then directly compared them to the averaged distances (ρ) and corresponding heuristic estimate of the standard error (σ), using whole-mtDNA sequences (excluding the mutations 16182C, 16183C, and 16519). We converted both ML and ρ mutational distances into years using the corrected molecular clock of [21].

We analysed the same dataset used to build the phylogenetic trees (196 N1a1b mitogenomes and 223 W with the exclusion of highly drifted Finnish mitogenomes, as in [49]) with BEAST v1.7 [52] to obtain Bayesian skyline plots (BSPs) [53], [54] of haplogroups N1a1b and W. We ran the program under the HKY substitution model (gamma-distributed rates) with a relaxed molecular clock (lognormal in distribution across branches and uncorrelated between them) for 100,000,000 iterations, with samples drawn every 10,000 Markov chain Monte Carlo (MCMC) steps, after a discarded burn-in of 10,000,000 steps, as in [55]. We considered haplogroups N1a1b, I and W as a whole and their major subclades monophyletic in the analyses. We visualized the BSPs obtained in plots with Tracer v1.5 and then converted them to Excel graphs by using a generation time of 25 years, as in [56]. We evaluated geographic distributions of both haplogroups I and W in a large dataset of more than 40,000 (published and unpublished) control-region (mostly limited to HVS-I, the first hypervariable segment) data from ∼100 populations, and assessed their geographic origin, haplogroup classification and haplotypes. We built spatial frequency distribution plots with the program Surfer 9 (Golden Software). We assigned the most likely source region for major clades in the whole-mtDNA tree on the basis of sample distribution among the subclades, following the same approach as in [22].

## Results

**Figure 1:**
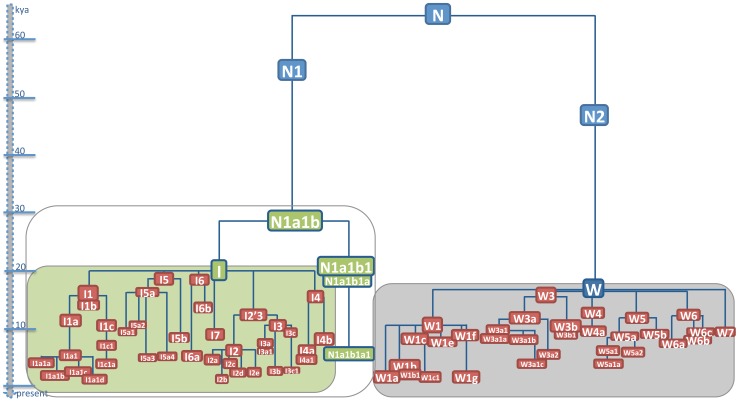
Phylogenetic tree of haplogroups N1a1b and W. This schematic representation is based on 196 N1a1b and 223 W mitogenomes whose phylogenetic relationships are illustrated in detail in Figure S1 and Figure S2. The phylogenetic connections between N1a1b and W are also shown. Approximate ages can be inferred from the scale. For haplogroups N1a1b and W, they correspond to the ML ages in Table 1 while previously reported ML ages were employed for nodes N, N1 and N2 [3].

The phylogenetic relationships of the 196 N1a1b (192 I and four representatives of its rare sister clade N1a1b1) and 223 W mitogenomes are depicted in detail in Figure S1 and Figure S2, while schematic trees are outlined in Figure 1. Additional information concerning the geographic and ethnic origin (when available) of each mitogenome is provided in Table S1 and Table S2 in File S1. Ages of N1a1b and W haplogroups and subclades are listed in Tables 1 and 2, respectively; overall values obtained were comparable across the two calculation methods employed (maximum likelihood (ML) and ρ). Haplogroups N1a1b1, I and W all descend from haplogroup N which, dating back to ∼60 kya [49], differentiated during the earliest phases of the out-of-Africa exit. The N1a1b ancestral node, ancestral to both haplogroups N1a1b1 and I, is nested deeply within haplogroup N1, passing through a series of intermediate nodes (Figure S1). Haplogroup N1a1b1 (former N1e), first defined by [57], is extremely rare and encompasses only four mitogenomes, one from Siberia and three from the Near East, including our new sequence from Iran (#194_Tor817). The introduction of this new mitogenome allows a better definition of the N1a1b1 clade, which radiates from the N1a1b root with the mutational motif 143-710-10790 and is subdivided into at least two newly defined nested subclades, namely N1a1b1a and N1a1b1a1.

**Table 1:** Molecular divergence and age estimates (maximum likelihood and ρ) for haplogroups N1a1b1 and I and their subclades.

		All Nucleotide Substitutions							
Haplogroup	*n* a	ML^b^	SE	Age (ky)^c^	95% CI (ky)	ρ	σ	Age (ky)^c^	95% CI (ky)
N1a1b	196	10.3	0.9	28.6	{23.5; 33.9}	10.7	3.1	29.8	{12.2; 48.5}
>N1a1b1	4	7.7	1.1	21.1	{14.9; 27.5}	8.5	3.0	23.3	{6.9; 41.1}
>>N1a1b1a	3	7.1	0.9	19.3	{14.6; 24.2}	7.0	3.2	19.0	{1.8; 37.8}
>>>N1a1b1a1	2	2.3	0.9	5.9	{1.3; 10.6}	2.0	1.0	5.2	{0.1; 10.5}
>I	192	7.4	0.3	20.1	{18.4; 21.9}	8.7	1.2	23.8	{16.9; 30.9}
>>I1	70	6.1	0.3	16.3	{14.6; 18.0}	8.7	3.2	23.9	{6.6; 42.7}
>>>I1a	45	4.4	0.3	11.6	{9.9; 13.3}	7.0	4.4	18.9	{−4.2; 44.9}
>>>>I1a1	41	1.9	0.1	4.9	{4.2; 5.6}	2.2	0.3	5.9	{4.3; 7.4}
>>>>>I1a1a	19	1.5	0.1	3.8	{3.3; 4.4}	1.7	0.1	4.4	{3.6; 5.1}
>>>>>I1a1b	4	0.5	0.2	1.4	{0.5; 2.2}	0.5	0.1	1.3	{0.7; 1.9}
>>>>>I1a1c	4	1.0	0.2	2.5	{1.3; 3.7}	1.3	0.7	3.2	{−0.2; 6.8}
>>>>>I1a1d	2	0.7	0.2	1.8	{1.0; 2.6}	1.5	0.8	3.9	{0.1; 7.8}
>>>I1b	11	5.0	0.4	13.4	{11.3; 15.5}	4.9	0.7	13.1	{9.3; 17.0}
>>>I1c	6	3.9	0.4	10.3	{8.4; 12.2}	5.3	2.7	14.3	{0.3; 29.4}
>>>>I1c1	5	2.7	0.3	7.2	{5.4; 9.0}	3.6	1.7	9.5	{0.8; 18.7}
>>>>>I1c1a	4	1.5	0.3	4.0	{2.5; 5.4}	1.8	0.4	4.6	{2.3; 6.8}
>>I5	29	6.8	0.3	18.4	{16.4; 20.3}	8.2	2.4	22.6	{9.1; 36.9}
>>>I5a	24	5.9	0.4	16.0	{14.0; 17.9}	6.8	1.5	18.4	{10.3; 26.9}
>>>>I5a1	9	3.5	0.4	9.2	{7.1; 11.3}	3.6	0.7	9.4	{5.5; 13.4}
>>>>I5a2	10	4.6	0.4	12.3	{10.2; 14.4}	5.4	3.3	14.5	{−2.9; 33.6}
>>>>>I5a2a	8	0.6	0.1	1.6	{1.0; 2.1}	0.6	0.1	1.6	{1.2; 2.0}
>>>>I5a3	2	1.8	0.4	4.8	{2.8; 6.8}	2.0	1.0	5.2	{0.1; 10.5}
>>>>I5a4	3	2.1	0.4	5.6	{3.5; 7.8}	2.7	1.1	7.0	{1.3; 12.9}
>>>I5b	2	3.3	0.5	8.8	{6.3; 11.2}	3.5	1.8	9.2	{0.2; 18.8}
>>I6	4	6.8	0.4	18.4	{16.2; 20.6}	8.5	3.3	23.3	{5.6; 42.6}
>>>I6a	2	2.0	0.3	5.3	{3.5; 7.0}	2.5	1.3	6.5	{0.1; 13.2}
>>>I6b	2	4.9	0.5	13.1	{10.4; 15.8}	5.5	2.8	14.8	{0.3; 30.4}
>>I7	2	3.5	0.5	9.1	{6.3; 11.9}	3.0	1.5	7.9	{0.2; 16.0}
>>node 152	65	5.8	1.2	15.5	{8.8; 22.5}	5.5	1.7	14.9	{5.8; 24.4}
>>>I2'3	64	4.7	0.4	12.6	{10.4; 14.7}	4.6	0.7	12.2	{8.2; 16.2}
>>>>I2	46	2.6	0.2	6.8	{6.0; 7.6}	3.4	0.2	9.1	{8.0; 10.2}
>>>>>I2a	10	1.8	0.2	4.7	{3.8; 5.7}	2.3	0.4	6.0	{3.9; 8.2}
>>>>>>I2a1	4	1.3	0.2	3.2	{2.1; 4.4}	2.0	0.8	5.2	{1.4; 9.2}
>>>>>I2b	4	0.7	0.2	1.7	{0.5; 2.9}	0.8	0.2	1.9	{1.0; 2.9}
>>>>>I2c	6	1.8	0.2	4.7	{3.6; 5.8}	1.5	0.3	3.9	{2.3; 5.5}
>>>>>I2d	3	1.1	0.4	3.0	{1.1; 4.8}	1.0	0.3	2.6	{0.9; 4.3}
>>>>>I2e	2	1.2	0.3	3.1	{1.4; 4.8}	1.0	0.5	2.6	{0.1; 5.2}
>>>>I3	18	4.0	0.3	10.6	{8.8; 12.4}	3.9	0.6	10.3	{7.3; 13.4}
>>>>>I3a	10	2.8	0.2	7.4	{6.1; 8.7}	2.6	0.3	6.8	{5.2; 8.4}
>>>>>>I3a1	2	2.3	0.3	6.1	{4.7; 7.5}	3.5	1.8	9.2	{0.2; 18.8}
>>>>>I3b	2	1.0	0.3	2.6	{1.1; 4.2}	1.0	0.5	2.6	{0.1; 5.2}
>>>>>I3c	3	3.5	0.3	9.4	{7.6; 11.2}	6.0	2.9	16.2	{0.9; 32.7}
>>I4	19	5.6	0.5	15.1	{12.3; 18.0}	3.7	1.1	9.9	{4.3; 15.7}
>>>I4a	17	2.4	0.2	6.4	{5.4; 7.4}	2.4	0.3	6.3	{5.0; 7.7}
>>>>I4a1	6	2.2	0.2	5.7	{4.7; 6.7}	3.2	0.6	8.3	{5.3; 11.5}
>>>I4b	2	3.2	0.5	8.4	{5.8; 10.9}	3.5	1.8	9.2	{0.2; 18.8}

a Number of mtDNA sequences.

b Maximum likelihood molecular divergence.

c Using the corrected molecular clock proposed by [21].

**Table 2:** Molecular divergence and age estimates (maximum likelihood and ρ) for haplogroup W and its subclades.

		All Nucleotide Substitutions							
Haplogroup	*n* a	ML^b^	SE	Age (ky)^c^	95% CI (ky)	ρ	σ	Age (ky)^c^	95% CI (ky)
W	223	6.2	0.5	16.8	{14.2; 19.5}	6.8	0.8	18.4	{14.1; 22.8}
>W1	93	3.9	0.3	10.4	{9.0; 11.9}	4.0	0.4	10.7	{8.5; 12.9}
>>W1a	25	0.6	0.1	1.6	{1.2; 2.0}	0.7	0.0	1.8	{1.5; 2.0}
>>W1b	15	1.1	0.2	2.8	{1.9; 3.6}	1.6	0.6	4.2	{1.0; 7.4}
>>>W1b1	11	0.8	0.1	2.0	{1.3; 2.6}	1.0	0.1	2.6	{2.0; 3.2}
>>W1e	5	1.6	0.4	4.2	{2.4; 6.1}	2.4	1.4	6.3	{−1.1; 14.0}
>>W1f	3	2.7	0.5	7.2	{4.8; 9.6}	2.7	1.6	7.0	{−1.0; 15.4}
>>119 node	27	3.3	0.2	8.6	{7.4; 9.8}	4.1	0.6	11.0	{7.6; 14.5}
>>>W1c	16	3.0	0.2	7.9	{6.8; 9.0}	4.4	0.7	11.8	{7.9; 15.8}
>>>>W1c1	6	1.8	0.2	4.6	{3.6; 5.7}	2.3	0.6	6.1	{3.2; 9.0}
>>W1g	3	0.8	1.3	2.0	{−4.6; 8.9}	0.7	0.4	1.7	{−0.5; 4.0}
>194 node	119	6.2	0.3	16.8	{15.0; 18.7}	7.4	0.9	20.3	{15.5; 25.2}
>>W3	45	5.5	0.3	14.8	{13.0; 16.6}	5.8	1.2	15.5	{8.9; 22.5}
>>>W3a	33	4.4	0.3	11.8	{10.1; 13.6}	4.8	1.0	12.7	{7.4; 18.2}
>>>>W3a1	29	3.7	0.2	9.8	{8.7; 10.9}	3.8	0.2	10.0	{8.8; 11.3}
>>>>>W3a1a	5	3.5	0.2	9.1	{8.0; 10.3}	5.8	1.4	15.6	{8.0; 23.5}
>>>>>>W3a1a1	2	3.2	0.2	8.3	{7.1; 9.5}	7.0	3.5	19.0	{0.4; 39.5}
>>>>>>W3a1a2	2	2.4	0.3	6.3	{4.6; 8.0}	3.5	1.8	9.2	{0.2; 18.8}
>>>>>W3a1b	5	3.3	0.2	8.7	{7.5; 9.9}	4.0	1.2	10.6	{4.3; 17.2}
>>>>>W3a1c	3	1.4	0.3	3.7	{2.2; 5.2}	1.3	0.4	3.5	{1.2; 5.8}
>>>>W3a2	3	2.1	1.7	5.6	{−3.0; 14.8}	2.7	1.6	7.0	{−1.0; 15.4}
>>>W3b	11	3.8	0.3	10.0	{8.2; 11.8}	3.7	0.7	9.9	{6.4; 13.4}
>>>>W3b1	3	0.4	0.2	1.0	{0.2; 1.8}	0.3	0.1	0.9	{0.3; 1.4}
>>W4	14	4.6	0.4	12.2	{9.9; 14.7}	4.6	0.8	12.2	{7.7; 16.8}
>>>W4a	8	3.7	0.4	9.8	{7.6; 12.0}	3.8	0.9	9.9	{5.1; 14.8}
>>W5	29	4.6	0.3	12.2	{10.5; 14.0}	5.8	2.3	15.6	{3.6; 28.4}
>>>W5a	24	3.2	0.3	8.4	{6.9; 9.8}	4.0	1.2	10.6	{4.2; 17.2}
>>>>W5a1	18	2.7	0.2	7.1	{5.9; 8.4}	3.4	1.0	9.1	{3.7; 14.7}
>>>>>W5a1a	16	2.1	0.2	5.5	{4.5; 6.6}	2.4	0.3	6.4	{4.9; 7.8}
>>>>>>W5a1a1	5	1.6	0.2	4.1	{3.0; 5.2}	2.2	0.6	5.7	{2.6; 8.9}
>>>>>>>W5a1a1a	2	0.4	0.1	1.0	{0.3; 1.7}	0.5	0.3	1.3	{0.0; 2.6}
>>>>W5a2	5	2.0	0.3	5.1	{3.7; 6.5}	2.0	0.5	5.2	{2.7; 7.7}
>>>W5b	4	3.5	0.4	9.3	{7.3; 11.4}	4.3	1.9	11.3	{1.2; 22.0}
>>W6	29	4.7	0.3	12.6	{10.8; 14.5}	5.4	1.1	14.4	{8.3; 20.7}
>>>16192 node	26	4.2	0.3	11.2	{9.8; 12.6}	4.3	0.4	11.6	{9.5; 13.6}
>>>>W6a	5	1.9	0.4	5.0	{3.1; 6.8}	1.6	0.6	4.2	{1.3; 7.1}
>>>>W6b	6	3.1	0.3	8.1	{6.4; 9.7}	4.3	1.1	11.5	{5.6; 17.6}
>>>W6c	2	3.6	0.4	9.6	{7.7; 11.4}	5.0	2.5	13.4	{0.3; 27.5}
>W7	4	3.7	0.7	9.9	{6.0; 14.0}	2.8	1.2	7.2	{1.1; 13.6}

a Number of mtDNA sequences.

b Maximum likelihood molecular divergence.

c Using the corrected molecular clock proposed by [21].

**Figure 2:**
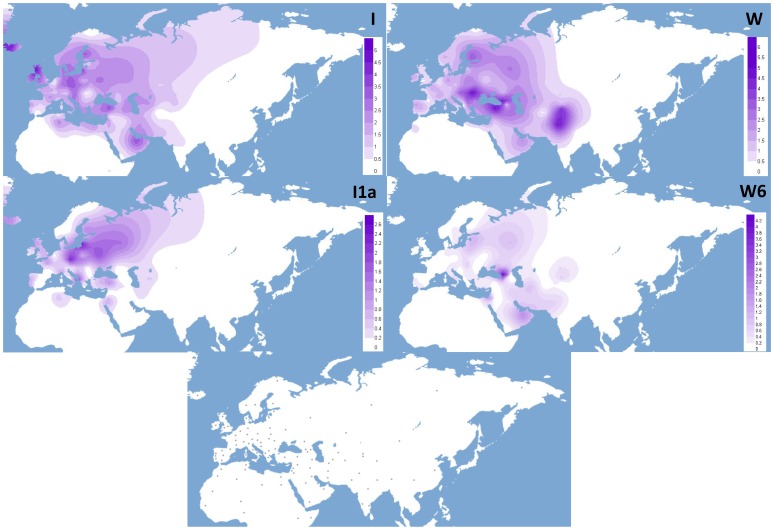
Spatial frequency distribution of haplogroups I and W and the sub-clades I1a and W6. Note that different frequency scales (%) were used in the maps. The dots in the lower map indicate the geographic location of the population samples included in the survey (Table S3 in File S1).

Haplogroup I encompasses the remaining 192 mitogenomes, including individuals from Africa, the Caucasus, the Near East and Europe. N1a1b dates to about 29 ky, suggesting a pre-LGM origin, while haplogroups N1a1b1 and I show very similar coalescence ages of 21.1 and 20.1 ky, respectively, during the span of the LGM (∼26–19 kya). Haplogroup I deviates by the mutational motif 10034–16129 from the N1a1b node and gives rise to at least seven major branches (I1–I7) which vary in terms of number of included samples, geographic distribution and even coalescence age (as outlined in Figure 1). Haplogroup I1, dating ∼16 ky, arose soon after haplogroup I during the Late Glacial pre-warming period and has been found mainly in Europe and the Near East, but also occasionally North Africa and the Caucasus. Among the subclades of I, I1 is both the most frequent in our tree, encompassing 70 mitogenomes out of 192, and also the most highly dissected, with at least three major subclades, I1a–c – all dating during the warming period ∼10–13 kya – with numerous nested subclades. In particular, the I1a subclade, encompassing 45 I1 samples and characterized by high internal haplotype diversity, is defined by the stable mutational motif 3447-8616T-16172 from the I1 root. The 16172 variant is located in HVS-I of the control region and we were therefore able to evaluate its distribution, in the context of the entire mutational motif of subclade I1a, in a large dataset of control-region sequences (Table S3 in File S1) to build I1a frequency maps (Figure 2). This distribution analysis revealed that frequency peaks of I1a (∼2.8%) are mainly localized in Europe, particularly in north-eastern Europe, with very low frequency values elsewhere (Table S3 in File S1).

Haplogroups I2 and I3 descend from the common root I2’3, which, in addition to the transition at position 207, shares a variant at position 152 from the root node of haplogroup I with a single mtDNA from Tanzania. However, this is an extremely mutable position, and for this East African mitogenome a possible affiliation within clades I2 or I3 – due to several reversions – should not be ruled out. The tree suggests that both haplogroups I2 and I3 might have arisen during the Holocene, with coalescence ages of ∼6.8 and 10.6 ky (with ML), respectively, and they show a star-like pattern with at least five subclades within I2 and three (including the two newly defined, *i.e.* I3b and I3c) within the less frequent I3. In our phylogeny, most representatives of both clades are from Europe, with only a few from the Near East, and notably subclades I2a and I2b are only seen in northern Europe.

This analysis also helps us to decipher the phylogenetic relationships of the subclades I4, I5, and I6, dating to the pre-warming or early warming period. I4 splits early into two subclades named I4a and I4b (newly defined) and encompasses samples from Europe, the Near East and the Caucasus. I5 is the second most frequent clade in the phylogeny (29 mtDNAs), and is mainly from Europe and the Near East (solely from the Near East in the case of subclades I5a2a and I5b, and almost solely from Europe for I5a1). In contrast, subclade I6 is very rare and has been found in only four subjects, mainly from the Near East. Even rarer is the newly defined subclade I7, which has been defined solely on the basis of two mitogenomes (from the Caucasus and the Near East) sharing a very stable mutational motif (Figure S1). This scarce clade, despite descending directly from the root of haplogroup I, has a Holocene age of ∼9.1 ky. In addition to the seven subclades of I, three mitogenomes (#100–102 in Figure S1; two from Somalia and one from Iran) are currently paraphyletic and are classified as I*.

Haplogroup W, represented by 223 full DNA genomes, is defined by the mutational motif 195@-204-207-1243-3505-5460-8251-8994-11947-15884C-16292 against the root of haplogroup N2, from which it descends directly, with no intermediates (Figure S2). Its coalescence age is slightly younger than haplogroups I and N1a1b1, dating to ∼17 kya, during the pre-warming period. Haplogroup W’s geographic distribution mostly overlaps with that of haplogroup I, but W reaches frequency peaks of ∼6% in northern India and in few regions of the Caucasus and the Near East, with further sporadic high frequency values in Eastern Europe (*e.g.* Romania, 6.5%) and Northern Europe (*e.g.* Finland, 4%), most likely due to founder effects (Table S3 in File S1, Figure 2).

Similar to haplogroup I, haplogroup W can be subdivided into at least six distinct subclades (W1, W3–7), with numerous and diversified internal branches (Figure 1). The W1 subclade, encompassing 93 out of 223 W mtDNAs, is the most frequent clade in the W tree (Figure S2). Dating to the early Holocene at ∼10.4 kya, it is defined by a single coding-region transition from the root of haplogroup W and it has quite a star-like structure, with at least six subclades (W1a, b, c, e, f, g) characterized by varying ages and geographic distributions. W1a and W1b (including W1b1), dating to 1.6 and 2.8 ky respectively, show strong signs of founder effects, comprising mitogenomes almost exclusively from Finland and northern Europe (Table S2 in File S1). The third most frequent W1 subclade is W1c, which dates to ∼8 kya and comprises individuals from Europe, the Near East, the Caucasus and India in our phylogeny (Figure S2, Table S2 in File S1), mainly arranged in a star-like shape.

Subclade W3, with an age of ∼15 ky, is the most ancient W lineage and also includes the most geographically heterogeneous collection of samples (spanning from Europe, including Russian regions, to North Africa, Caucasus, the Near East, Mongolia and the Indian Subcontinent), virtually covering the whole distribution range of haplogroup W. Unfortunately, W3 is defined solely by a coding-region transition at position 1406, thus making impossible a better evaluation of its true spatial distribution using the control-region database. W3 splits into two subclades, W3a and W3b, dating back to the late Pleistocene/early Holocene: ∼12 and 10 kya, respectively. There is also a single basal lineage in an individual from the South Caucasus. W3a further splits into W3a1 (∼9.8 ky) and W3a2 (∼5.6 ky), of which W3a1 is the more frequent and includes at least three subclades, with W3a1b encompassing only mtDNAs from India, and a number of paraphyletic lineages.

Subclades W4, W5 and W6, despite bearing very similar coalescence ages ∼12.2–12.6 ky, show different geographic distributions and structures (Figure S2). While W4 spans Europe, the Near East, Mongolia and India, W5, despite being dissected into the newly defined sub-clades W5b and W5a, seems to be mainly diffused in Europe, with a single basal mtDNA from a Moroccan Berber individual directly descending from the W5 root. W6 is defined by the combination of two coding-region transitions (4093 and 8614) and the stable control-region variant at position 16325, which allowed us to search for its control-region mutational motif in our database (Table S3 in File S1) and present its distribution map (Figure 2). Haplogroup W6 is most frequent in the Caucasus - with a peak of ∼5.3% in Georgia - and the Levant, widespread within Europe but at frequencies <1% (with the exception of Estonia), very rare in India and central Asia, and absent from Africa.

Finally, following PhyloTree classification, haplogroup W divides into subclade W7, which dates to ∼10 ky and is represented by only four mtDNAs, mostly, except one from Armenia, of unknown geographic origin. However, W7 is defined solely by a variant at the unstable control-region position 185 (three occurrences in our tree), thus it may not constitute a true clade. Moreover, at least nine paraphyletic mitogenomes (mainly from the Near East) do not fall within any of the seven major sub-clades of haplogroup W. In particular, the Italian sample #223, bearing a single reversion of the defining control-region variant at position 16292, might even possibly radiate before the root of the entire haplogroup W. However, the hypothesis of a founder haplotype from central Italy remains enigmatic and would need further evidence to be properly supported.

**Figure 3:**
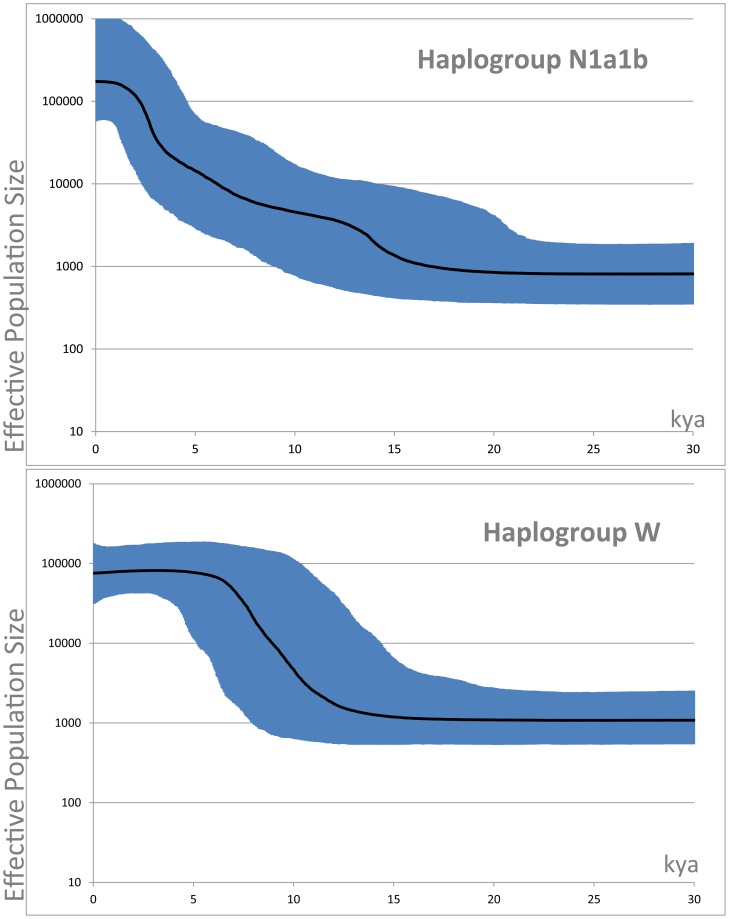
Bayesian Skyline Plots (BSPs) of haplogroups N1a1b and W. Hypothetical effective population sizes through time are based on the mitogenomes listed in Table S1 and Table S2 (File S1).

The Bayesian Skyline Plots (BSPs) of haplogroups N1a1b and W indicate two major expansion periods - at ∼11–13 and from ∼7 kya – for haplogroup N1a1b, while we detected a major increment starting from ∼13 kya for haplogroup W (Figure 3).

## Discussion

In the last ten years, the availability of a growing number of complete mitogenomes (more than 18,000 in [47]) has dramatically improved the worldwide human mtDNA phylogeny [48]. Many of the novel subclades are characterized by more distinct geographical distributions than the deeper clades from which they derive, thus allowing inferences on demographic events that not only occurred more recently but at regional rather than continental level (*e.g.* [19], [22]). In this study, we aimed to define the internal variation of haplogroups N1a1b and W, which are rather uncommon and were not well-sampled in random population surveys. Moreover, despite their infrequent occurrence, both N1a1b and W have extremely wide distribution ranges encompassing the whole of Western Eurasia and North Africa, implying that extensive hidden substructure remained to be uncovered for both haplogroups. Our data confirm this scenario, bringing to light numerous novel subclades as well as improving the phylogenetic resolution of those already known.

Our data confirm that N1a1b1 and I coalesce at very similar times (21.1 and 20.1 kya, respectively) and their common molecular ancestor, corresponding to the N1a1b node, arose 28.6±5.2 kya. Haplogroup N1a1b1, even if very rare, has been found only in Asia with a deep internal split at node N1a1b1a (dated at ∼19.3 kya) which divides one single mitogenome from Russian North Asia (Siberia) and the remaining three Near Eastern (Iranian) individuals. Haplogroup I has a more widespread distribution, but with peaks of frequency in the Near East. Therefore, the most parsimonious scenario is that both haplogroups N1a1b1 and I arose in the Near East during the LGM period. This conclusion is supported by the phylogeny of haplogroup I. All of the subclades of haplogroup I, and especially the Late Glacial subclades (I1, I4, I5, and I6), include mitogenomes from the Near East. Like the deeper subclades of I, haplogroup W also dates to the Late Glacial period, ∼17 kya, and most of its subclades (W3–6) differentiated during the warming period (12–15 kya). Moreover, the distribution of W, with frequency peaks in India, the Near East and the Caucasus, as well as the presence of numerous basal Near Eastern lineages in the W tree (also dating to the Late Glacial), might suggest an origin in the Near East as well, with a subsequent very rapid spread into Europe.

Comparing phylogeographic data from other lineages of Near Eastern origin, the overall age estimates for N1a1b1, I, and W haplogroups appear very similar to those previously reported for major subclades of J and T, two among the most frequent haplogroups in Europe and the Near East [22]. These major subclades were recently identified as signals of dispersals into Europe from a Near Eastern refuge area, after the peak of the last glaciation, ∼19 kya [22]. This scenario may be paralleled in the history of haplogroups N1a1b1, I and W, with dispersals of haplogroups I and W into Europe during the Late Glacial period, ∼18–12 kya, signalled in particular by subclades I1, I2’3, I5, W3, W4 and W5, and by W1 in the immediate postglacial period, ∼10–11 kya. Thus important expansions of I and W occurred in parallel with Late Glacial and postglacial climatic improvements, several millennia before the European Neolithic.

It seems likely that Late Glacial and postglacial improvements in climate were fundamental to the dispersal of numerous other mtDNA lineages not only in the Near East and Europe, but also in Africa, Asia, the Pacific and the Americas [3], [21], [54], [55], [58]–[64], and even some lineages previously thought to be markers for Neolithic expansions have now been recognized as signalling Late Palaeolithic and/or Mesolithic diffusion events [22]. This represents a significant step forward in the century-long debate concerning the relative genetic contribution of Palaeolithic *versus* Neolithic to the current gene pool of modern Europeans. Now, a still unresolved fundamental question in understanding the genetic makeup of modern Europeans is what exactly happened in the time span of several thousands of years between the Late Palaeolithic/Mesolithic expansions and the arrival of agriculture in the different parts of Europe. The first clear consequence of the scenario described above is that since the European genetic pool was largely defined before Neolithic times, major haplogroups already present in Europe during the Palaeolithic were most probably involved in subsequent gene flows linked to the advent and expansion of agriculture. Therefore, we need to distinguish between lineages that arrived from the Near East with agriculture – which appear to be few, in the extant mtDNA pool – and those which may have dispersed and expanded within Europe, carrying agriculture from one region to another. In the case of the haplogroup I and W phylogenies, signs of the diffusion of agriculture and pastoralism within Europe may be evident in those I and W subclades which date to the European Neolithic period and are restricted to Europe, particularly starlike examples such as I1a1 (as already suggested by [49]) and I2, and possibly also I1c1, I3 and W5a. These are reflected in the more recent of the two bursts of growth starting from ∼7 kya in the N1a1b BSP of Figure 3, while the major expansion of the entire haplogroup W started during the Late Glacial period, decreasing gradually during the Neolithic (Figure 3). It is worth noting that the autosomal STRUCTURE analyses for Europe carried out by Behar et al. [65] seem to suggest a very substantial indigenous (*i.e.* non-Near Eastern) component, along with two potentially Near Eastern components, which could perhaps correspond to distinct Late Glacial and Neolithic dispersals from the Near East. Recent simulation work attempting to interpret autosomal patterns also suggests that any Neolithic immigration is likely to have been very minor [66]. However, with extant evidence we can only estimate (at best) the degree of present-day impact of each dispersal, rather than the scale of the dispersal as it was at the time.

The direct comparison of ancient and modern DNA samples, allowing a diachronic view of human history, can be an important test of inferences based on data from extant populations. Having improved the resolution of the N1a1b and W phylogenies, we were then able to re-evaluate the published I and W control-region haplotypes from ancient specimen (Table 3) in the context of the modern variation of I and W mitogenomes (Figure S1 and Figure S2). Since only information relative to the HVS-I is available, most of the ancient I and W mtDNAs bear basal and/or common haplotypes, which could not be further classified within any subclade. However, a few informative cases were identified. A Spanish middle Neolithic sample [40] bearing the haplogroup I control-region motif 16264-16270-16311-16319-16362 (from the root of I) (Table 3) can now be classified within I1c1. The identification of this sample has already been interpreted as indicating genetic continuity in the Iberian Peninsula since the Neolithic period and (more contentiously, given the paucity of Mesolithic evidence from Iberia) that the diffusion of agriculture followed a demic model in the Mediterranean area [40]. We found that the same I1c1 haplotype is shared by five mitogenomes in our phylogeny (#60-64 in Figure S1), of which four are of unknown geographic/ethnic origin but one (sample #64, sequenced in the present study) was of North Italian origin. This result provides a further confirmation of our findings, based on the analysis of haplogroup I phylogeny, that (i) subclade I1c1 was likely a marker of Neolithic dispersal in Europe (rather than, for example, having been brought from the Near East much more recently by the ancestors of Ashkenazi Jews, some of whom carry this lineage [67], [68]) and (ii) the distribution and age might support a demic model of Neolithic diffusion in the Mediterranean area. Similarly, a probable member of haplogroup W3 in the same Spanish Neolithic sample [40], sharing the haplotype 16292-16295-16304 (against the root of N) with a mitogenome from Azerbaijan (sample #127) in our phylogeny), may point to Neolithic dispersal from the Near East into Europe.

**Table 3:** List of ancient specimen belonging to mtDNA haplogroups I and W and control-region haplotypes.

Country	Site	Date (BC)	Cultural Period	Haplogroup	Control-Region Haplotype^1^	Sequence Range	Reference
Spain	Paternanbidea, Navarra	6090–5960	Neolithic	I	16129 16233	HVS-I, HVS-II	[71]
Spain	Cami de Can GrauGranollers, Barcelona	3500–3000	Neolithic	I1c1	(16129) 16223 16264 16270 16311 16319 16362	HVS-I	[40]
Germany	Kromsdorf	2600–2500	Late Neolithic - Bell Beaker culture	I1a1	16129 16172 16223 16311 16391 73199 203 204 250 263	15995–16429, 34–287	[72]
Germany	Eulau	2563–2465	Late Neolithic - Corded Ware culture	I	16129 16223 16391	15997–16409	[73]
Germany	Esperstedt	2050–1800	Late Neolithic - Unetice culture	I	16129 16223 16278 16311 16391	15997–16409	[70]
Germany	Esperstedt	2050–1800	Late Neolithic - Unetice culture	I	16086 16129 16223 16391	15997–16409	[70]
Kazakhstan	Vodokhranilische	1400–1000	Bronze Age - Kurgan culture	I	16129 16223 16294	HVS-I	[74]
Germany	Derenburg Meerenstieg II	5000	Early Neolithic - LBK culture	W	16093 16223 16292	15997–16409	[42]
Germany	Derenburg Meerenstieg II	5000	Early Neolithic - LBK culture	W	16093 16223 16292	15997–16409	[42]
Spain	Cami de Can GrauGranollers, Barcelona	3500–3000	Neolithic	W	16223 16292 16295 16304	HVS-I	[40]
Germany	Kromsdorf	2600–2500	Late Neolithic - Bell Beaker culture	W5a?	16223 16292 16362 73 189 194 195204 207 263	15995–16429, 34–287	[72]
Germany	Esperstedt	2700–2000	Late Neolithic - Corded Ware culture	W6	16192 16223 16292 16325	15997–16409	[70]
Germany	Esperstedt	2050–1800	Late Neolithic - Unetice culture	W	16147G 16223 16292	15997–16409	[70]
Kazakhstan	Zevakinskiy	800–700	Bronze/Iron Age	W	16223 16292	HVS-I	[74]

1 Control-region haplotypes are from the root of haplogroup N.

With the advent of reliable ancient DNA studies, attention is starting to focus on subsequent events in European prehistory. A German specimen associated with the Late Neolithic Bell Beaker culture bears the mtDNA control-region variants (from the root of N) 16129-16172-16311-16391-73-199-203-204-250-263 (Table 3). The mutational motif 16172-203 classifies this sample within I1a1, another potential marker of the agricultural expansion in Europe. Considering that haplogroup I1a (Figure 3), from which subclade I1a1 derives, is mainly concentrated in Europe, with frequency peaks in Eastern Europe, it is possible that sub-clade I1a1, dated to about 5 kya in our phylogeny (Table 1), might be a marker of a late Neolithic diffusion from Central/Eastern Europe, perhaps associated with the Corded Ware, into Bell Beaker territory. This would also be consistent with the lack of haplogroup I1 thus far (apart from I1c) in any western European Neolithic or pre-Neolithic remains [69], and would testify to the importance of dispersals later than the early Neolithic in prehistoric Europe. Similarly, the European Neolithic subclade W5a has been detected in one Late Neolithic sample of the German Bell Beaker culture (Table 3), even though an accurate classification for this sample would require the analysis of at least one of the W5a-specific coding-region markers. Indeed, a German individual belonging to the Corded Ware culture has been shown to carry a W6 lineage [70]. As with I1a1, the age and distribution again makes an origin in the north-east European Neolithic, followed by dispersal westwards with the Late Neolithic, an attractive hypothesis.

## Supporting Information

Figure S1 **Maximum parsimony tree of 196 mitogenomes belonging to the sister haplogroups I and N1a1b1.**
(XLSX)

Figure S2 **Maximum parsimony tree of 223 mitogenomes belonging to haplogroup W.**
(XLSX)

File S1 **File containing Tables S1–S3.** Table S1. Origin and subclade affiliation of haplogroup N1a1b1 and I mitogenomes considered in this study. Table S2. Origin and subclade affiliation of haplogroup W mitogenomes considered in this study. Table S3. Percentage frequency distribution of haplogroups I and W and the subclades I1a and W6.
(DOCX)
