# Supplementary material for: Mitogenomes from Two Uncommon Haplogroups Mark Late Glacial/Postglacial Expansions from the Near East and Neolithic Dispersals within Europe
Source: PLoS One. 2013 Jul 31;8(7):e70492. doi: 10.1371/journal.pone.0070492 (PMC3729697; doi:10.1371/journal.pone.0070492)
Supplement: File S1 — File containing Tables S1–S3. Table S1. Origin and subclade affiliation of haplogroup N1a1b1 and I mitogenomes considered in this study. Table S2. Origin and subclade affiliation of haplogroup W mitogenomes considered in this study. Table S3. Percentage frequency distribution of haplogroups I and W and the subclades I1a and W6. (DOCX) [file pone.0070492.s003.docx]

**SUPPLEMENTARY MATERIAL**

**Table S1.** Origin and sub-haplogroup affiliation of haplogroup N1a1b1 and I mitogenomes considered in this study.

| Sample # | Sequence ID^a^ | Haplogroup | Region | Country/Ethnicity | GenBank ID | Ref (provided by) | PubMed ID |
| --- | --- | --- | --- | --- | --- | --- | --- |
| 1 | Tor810 | I1a1a | Europe East | Poland | KF146236 | This Study |  |
| 2 | AY339503 | I1a1a | Europe North | Finland | AY339503 | [1] | 11349229 |
| 3 | AY339504 | I1a1a | Europe North | Finland | AY339504 | [1] | 11349229 |
| 4 | AY339502 | I1a1a | Europe North | Finland | AY339502 | [1] | 11349229 |
| 5 | AY339505 | I1a1a | Europe North | Finland | AY339505 | [1] | 11349229 |
| 6 | AY339506 | I1a1a | Europe North | Finland | AY339506 | [1] | 11349229 |
| 7 | HG00369 | I1a1a | Europe North | Finland |  | 1000 Genomes Project |  |
| 8 | AY339508 | I1a1a | Europe North | Finland | AY339508 | [1] | 11349229 |
| 9 | AY339509 | I1a1a | Europe North | Finland | AY339509 | [1] | 11349229 |
| 10 | AY339507 | I1a1a | Europe North | Finland | AY339507 | [1] | 11349229 |
| 11 | JQ705140 | I1a1a |  |  | JQ705140 | [2] | 22482806 |
| 12 | Tor811 | I1a1a | Europe West | Italy, Sardinia | KF146237 | This Study |  |
| 13 | JQ245767 | I1a1a | Near East | Turkey | JQ245767 | [3] | 22284828 |
| 14 | JQ245749 | I1a1a | Europe East | Czech Republic | JQ245749 | [3] | 22284828 |
| 15 | JQ245748 | I1a1a | Europe East | Czech Republic | JQ245748 | [3] | 22284828 |
| 16 | JQ705378 | I1a1a |  |  | JQ705378 | [2] | 22482806 |
| 17 | JQ704013 | I1a1a |  |  | JQ704013 | [2] | 22482806 |
| 18 | JQ703652 | I1a1a |  |  | JQ703652 | [2] | 22482806 |
| 19 | JQ702939 | I1a1a |  |  | JQ702939 | [2] | 22482806 |
| 20 | JQ705889 | I1a1 |  |  | JQ705889 | [2] | 22482806 |
| 21 | FJ460562 | I1a1 | Africa North | Tunisia | FJ460562 | [4] | 19133286 |
| 22 | JQ705645 | I1a1 |  |  | JQ705645 | [2] | 22482806 |
| 23 | EF177414 | I1a1 | Europe West | Portugal | EF177414 | Pereira et al. | Direct Submission |
| 24 | HG00253 | I1a1b | Europe North | Great Britain |  | 1000 Genomes Project |  |
| 25 | JQ704690 | I1a1b |  |  | JQ704690 | [2] | 22482806 |
| 26 | JQ705595 | I1a1b |  |  | JQ705595 | [2] | 22482806 |
| 27 | JQ702470 | I1a1b |  |  | JQ702470 | [2] | 22482806 |
| 28 | JQ245802 | I1a1 | Africa North | Morocco | JQ245802 | [3] | 22284828 |
| 29 | GU123027 | I1a1c | Europe East | Russia | GU123027 | [5] | 20457583 |
| 30 | JQ702023 | I1a1c |  |  | JQ702023 | [2] | 22482806 |
| 31 | JQ702457 | I1a1c |  |  | JQ702457 | [2] | 22482806 |
| 32 | Tor813 | I1a1c | Europe East | Ukraine | KF146238 | This Study |  |
| 33 | Tor815 | I1a1 | Europe West | Italy, Piedmont | KF146239 | This Study |  |
| 34 | JQ705189 | I1a1d |  |  | JQ705189 | [2] | 22482806 |
| 35 | JQ702342 | I1a1d |  |  | JQ702342 | [2] | 22482806 |
| 36 | JQ702519 | I1a1 |  |  | JQ702519 | [2] | 22482806 |
| 37 | JQ702820 | I1a1 |  |  | JQ702820 | [2] | 22482806 |
| 38 | JQ701900 | I1a1 |  |  | JQ701900 | [2] | 22482806 |
| 39 | JQ705025 | I1a1 |  |  | JQ705025 | [2] | 22482806 |
| 40 | JQ703835 | I1a1 |  |  | JQ703835 | [2] | 22482806 |
| 41 | JQ702882 | I1a1 |  |  | JQ702882 | [2] | 22482806 |
| 42 | JQ245746 | I1a | Europe East | Russia, Chuvash | JQ245746 | [3] | 22284828 |
| 43 | HM852839 | I1a | Near East | Iran | HM852839 | [6] | 21487439 |
| 44 | EU694173 | I1a | Unknown | - | EU694173 | Family Tree DNA | Direct Submission |
| 45 | HM454265 | I1a | Caucasus South | Armenia | HM454265 | Family Tree DNA | Direct Submission |
| 46 | JQ705376 | I1b |  |  | JQ705376 | [2] | 22482806 |
| 47 | EF556153 | I1b |  |  | EF556153 | [7] | 18446216 |
| 48 | FJ968796 | I1b | USA |  | FJ968796 | Family Tree DNA | Direct Submission |
| 49 | JQ704018 | I1b |  |  | JQ704018 | [2] | 22482806 |
| 50 | Tor821 | I1b | Europe West | Italy, Sardinia | KF146240 | This Study |  |
| 51 | Tor798 | I1b | Near East | Iran, Hormozgan | KF146241 | This Study |  |
| 52 | Tor803 | I1b | Near East | Iran, Khuzestan | KF146242 | This Study |  |
| 53 | FJ234984 | I1b | Caucasus South | Armenia | FJ234984 | Family Tree DNA | Direct Submission |
| 54 | Tor800 | I1b | Europe West | Italy | KF146243 | This Study |  |
| 55 | AY714041 | I1b | Asia South | India | AY714041 | [8] | 15467980 |
| 56 | AY195769 | I1b | Europe | Europe | AY195769 | [9] | 12509511 |
| 57 | HM156684 | I1 | Asia South | India | HM156684 | [10] | 21296687 |
| 58 | JQ245776 | I1 | Near East | Turkey | JQ245776 | [3] | 22284828 |
| 59 | JQ705932 | I1c |  |  | JQ705932 | [2] | 22482806 |
| 60 | JQ705364 | I1c1a |  |  | JQ705364 | [2] | 22482806 |
| 61 | EU564849 | I1c1a | Unknown | - | EU564849 | Family Tree DNA | Direct Submission |
| 62 | JQ705190 | I1c1a |  |  | JQ705190 | [2] | 22482806 |
| 63 | JQ702655 | I1c1a |  |  | JQ702655 | [2] | 22482806 |
| 64 | Tor818 | I1c1 | Europe West | Italy, Lombardy | KF146244 | This Study |  |
| 65 | Tor940 | I1 | Europe West | Italy | KF146245 | This Study |  |
| 66 | Tor809 | I1 | Europe West | Italy, Campania | KF146246 | This Study |  |
| 67 | JQ704077 | I1 |  |  | JQ704077 | [2] | 22482806 |
| 68 | JQ702472 | I1 |  |  | JQ702472 | [2] | 22482806 |
| 69 | JQ705840 | I1 |  |  | JQ705840 | [2] | 22482806 |
| 70 | JQ702567 | I1 |  |  | JQ702567 | [2] | 22482806 |
| 71 | JQ245781 | I5a2a | Near East | Yemen | JQ245781 | [3] | 22284828 |
| 72 | JQ245782 | I5a2a | Near East | Yemen | JQ245782 | [3] | 22284828 |
| 73 | JQ245783 | I5a2a | Near East | Yemen | JQ245783 | [3] | 22284828 |
| 74 | JQ245786 | I5a2a | Near East | Yemen | JQ245786 | [3] | 22284828 |
| 75 | JQ245784 | I5a2a | Near East | Yemen | JQ245784 | [3] | 22284828 |
| 76 | JQ245785 | I5a2a | Near East | Yemen | JQ245785 | [3] | 22284828 |
| 77 | JQ245733 | I5a2a | Near East | Dubai | JQ245733 | [3] | 22284828 |
| 78 | JQ245780 | I5a2a | Near East | Yemen | JQ245780 | [3] | 22284828 |
| 79 | JQ701894 | I5a2 |  |  | JQ701894 | [2] | 22482806 |
| 80 | NA12342 | I5a2 | Europe |  |  | 1000 Genomes Project |  |
| 81 | EU597573 | I5a1 | Near East | Israel, Bedouin | EU597573 | Hartmann et al. | Direct Submission |
| 82 | Tor814 | I5a1 | Europe West | Italy, Tuscany | KF146247 | This Study |  |
| 83 | JQ704768 | I5a1 |  |  | JQ704768 | [2] | 22482806 |
| 84 | JQ245807 | I5a1 | Europe East | Bulgaria | JQ245807 | [3] | 22284828 |
| 85 | JQ705096 | I5a1 |  |  | JQ705096 | [2] | 22482806 |
| 86 | Tor799 | I5a1 | Europe West | Italy, Sardinia | KF146248 | This Study |  |
| 87 | AF382007 | I5a1 | Europe West | Spain, Leon | AF382007 | [11] | 11553319 |
| 88 | EF660917 | I5a1 | Europe West | Italy | EF660917 | [12] | 17517629 |
| 89 | JQ704713 | I5a1 |  |  | JQ704713 | [2] | 22482806 |
| 90 | Tor527 | I5a3 | Europe West | Germany | JN415483 | [13] | 22879922 |
| 91 | JQ245772 | I5a3 | Near East | Turkey | JQ245772 | [3] | 22284828 |
| 92 | Tor819 | I5a4 | Europe West | Italy, Marche | KF146249 | This Study |  |
| 93 | HM852869 | I5a4 | Near East | Turkey | HM852869 | [6] | 21487439 |
| 94 | FJ348190 | I5a4 | Europe East | USA, Hutterite from Russia | FJ348190 | [14] | 19844259 |
| 95 | JQ245724 | I5 | Caucasus North | Ossetia | JQ245724 | [3] | 22284828 |
| 96 | HM852817 | I5b | Near East | Iran | HM852817 | [6] | 21487439 |
| 97 | Tor822 | I5b | Near East | Iran, Azerbaijan | KF146250 | This Study |  |
| 98 | Tor797 | I5 | Europe West | Italy, Marche | KF146251 | This Study |  |
| 99 | HQ658465 | I5 | Europe North | Germany | HQ658465 | Family Tree DNA | Direct Submission |
| 100 | JQ245791 | I | Africa East | Somalia | JQ245791 | [3] | 22284828 |
| 101 | JQ245792 | I | Africa East | Somalia | JQ245792 | [3] | 22284828 |
| 102 | Tor805 | I | Near East | Iran, Lorestan | KF146252 | This Study |  |
| 103 | AY245555 | I6a | Europe? |  | AY245555 | [15] | 16705548 |
| 104 | JQ705382 | I6a |  |  | JQ705382 | [2] | 22482806 |
| 105 | JQ245773 | I6b | Near East | Turkey | JQ245773 | [3] | 22284828 |
| 106 | HM852831 | I6b | Near East | Iran | HM852831 | [6] | 21487439 |
| 107 | JF298212 | I7 | Caucasus South | Armenia | JF298212 | Family Tree DNA | Direct Submission |
| 108 | Tor804 | I7 | Near East | Kuwait | KF146253 | This Study |  |
| 109 | HQ695930 | I2a | USA |  | HQ695930 | Family Tree DNA |  |
| 110 | AY339497 | I2a1 | Europe North | Finland | AY339497 | [1] | 11349229 |
| 111 | HG00329 | I2a1 | Europe North | Finland |  | 1000 Genomes Project |  |
| 112 | HQ724528 | I2a1 | Europe North | Ireland, County Clare | HQ724528 | Family Tree DNA | Direct Submission |
| 113 | JN411083 | I2a1 | Europe North | Ireland | JN411083 | Family Tree DNA | Direct Submission |
| 114 | HQ714959 | I2a | Europe North | Scotland | HQ714959 | Family Tree DNA |  |
| 115 | HQ326985 | I2a | USA |  | HQ326985 | Family Tree DNA | Direct Submission |
| 116 | JQ705921 | I2a |  |  | JQ705921 | [2] | 22482806 |
| 117 | JQ703910 | I2a |  |  | JQ703910 | [2] | 22482806 |
| 118 | JQ705175 | I2a |  |  | JQ705175 | [2] | 22482806 |
| 119 | AY339498 | I2b | Europe North | Finland | AY339498 | [1] | 11349229 |
| 120 | AY339500 | I2b | Europe North | Finland | AY339500 | [1] | 11349229 |
| 121 | AY339501 | I2b | Europe North | Finland | AY339501 | [1] | 11349229 |
| 122 | AY339499 | I2b | Europe North | Finland | AY339499 | [1] | 11349229 |
| 123 | GU294854 | I2 | Canada | Caucasian | GU294854 | Family Tree DNA | Direct Submission |
| 124 | JQ705187 | I2c |  |  | JQ705187 | [2] | 22482806 |
| 125 | JQ702253 | I2c |  |  | JQ702253 | [2] | 22482806 |
| 126 | JQ702163 | I2c |  |  | JQ702163 | [2] | 22482806 |
| 127 | JQ705666 | I2c |  |  | JQ705666 | [2] | 22482806 |
| 128 | JQ703024 | I2c |  |  | JQ703024 | [2] | 22482806 |
| 129 | HG00124 |  | Europe North | Great Britain |  | 1000 Genomes Project |  |
| 130 | GU122984 | I2 | Europe East | Russia | GU122984 | [5] | 20457583 |
| 131 | JQ703829 | I2d |  |  | JQ703829 | [2] | 22482806 |
| 132 | JQ245747 | I2d | Europe East | Czech Republic | JQ245747 | [3] | 22284828 |
| 133 | JQ705244 | I2d |  |  | JQ705244 | [2] | 22482806 |
| 134 | HQ287882 | I2 | Europe | Canada, Newfoundland | HQ287882 | [16] | 21326367 |
| 135 | Tor808 | I2 | Europe West | Italy, Marche | KF146254 | This Study |  |
| 136 | Tor812 | I2 | Europe East | Ukraine | KF146255 | This Study |  |
| 137 | JQ245744 | I2 | Caucasus North | Chechnya | JQ245744 | [3] | 22284828 |
| 138 | JQ245771 | I2 | Near East | Turkey | JQ245771 | [3] | 22284828 |
| 139 | JQ702284 | I2 |  |  | JQ702284 | [2] | 22482806 |
| 140 | JQ704705 | I2 |  |  | JQ704705 | [2] | 22482806 |
| 141 | JQ702191 | I2 |  |  | JQ702191 | [2] | 22482806 |
| 142 | JQ703106 | I2e |  |  | JQ703106 | [2] | 22482806 |
| 143 | JQ702578 | I2e |  |  | JQ702578 | [2] | 22482806 |
| 144 | Tor820 | I2 | Europe West | Italy, Piedmont | KF146256 | This Study |  |
| 145 | JQ703850 | I2 |  |  | JQ703850 | [2] | 22482806 |
| 146 | JQ701942 | I2 |  |  | JQ701942 | [2] | 22482806 |
| 147 | JQ704765 | I2 |  |  | JQ704765 | [2] | 22482806 |
| 148 | JQ705304 | I2 |  |  | JQ705304 | [2] | 22482806 |
| 149 | Tor816 | I2 | Europe West | Italy, Piedmont | KF146257 | This Study |  |
| 150 | FJ911909 | I2 | Europe North | England | FJ911909 | Family Tree DNA | Direct Submission |
| 151 | JQ705379 | I2 |  |  | JQ705379 | [2] | 22482806 |
| 152 | JQ704936 | I2 |  |  | JQ704936 | [2] | 22482806 |
| 153 | JQ705000 | I2 |  |  | JQ705000 | [2] | 22482806 |
| 154 | EU570217 | I2 | Europe North | Ireland | EU570217 | Family Tree DNA | Direct Submission |
| 155 | JQ702041 | I3a |  |  | JQ702041 | [2] | 22482806 |
| 156 | JQ245751 | I3a | Europe East | Greece | JQ245751 | [3] | 22284828 |
| 157 | Tor801 | I3a | Near East | Iran, Gilan | KF146258 | This Study |  |
| 158 | EU746658 | I3a | Europe West | France | EU746658 | Family Tree DNA | Direct Submission |
| 159 | HQ420832 | I3a1 | Europe West | France | HQ420832 | Family Tree DNA | Direct Submission |
| 160 | JQ704837 | I3a1 |  |  | JQ704837 | [2] | 22482806 |
| 161 | JQ702109 | I3a |  |  | JQ702109 | [2] | 22482806 |
| 162 | EU869314 | I3a | Europe North | England | EU869314 | Family Tree DNA | Direct Submission |
| 163 | Tor21 | I3a | Europe West | Italy | AY963586 | [17] |  |
| 164 | JQ702062 | I3a |  |  | JQ702062 | [2] | 22482806 |
| 165 | JQ705377 | I3 |  |  | JQ705377 | [2] | 22482806 |
| 166 | JQ703883 | I3 |  |  | JQ703883 | [2] | 22482806 |
| 167 | JQ702493 | I3 |  |  | JQ702493 | [2] | 22482806 |
| 168 | GU590993 | I3b | Europe North | Ireland | GU590993 | Family Tree DNA | Direct Submission |
| 169 | JQ702413 | I3b |  |  | JQ702413 | [2] | 22482806 |
| 170 | JQ702647 | I3c |  |  | JQ702647 | [2] | 22482806 |
| 171 | JQ703862 | I3c |  |  | JQ703862 | [2] | 22482806 |
| 172 | Tor806 | I3c | Europe West | Italy, Marche | KF146259 | This Study |  |
| 173 | EF184634 | I | Africa East | Tanzania | EF184634 | [18] | 17194802 |
| 174 | JQ702369 | I4a |  |  | JQ702369 | [2] | 22482806 |
| 175 | JQ705514 | I4a |  |  | JQ705514 | [2] | 22482806 |
| 176 | JN660158 | I4a | Caucasus South | Armenia | JN660158 | Family Tree DNA | Direct Submission |
| 177 | JQ705906 | I4a1 |  |  | JQ705906 | [2] | 22482806 |
| 178 | JQ705191 | I4a1 |  |  | JQ705191 | [2] | 22482806 |
| 179 | EF660987 | I4a1 | Europe West | Italy | EF660987 | [12] | 17517629 |
| 180 | HM804481 | I4a1 | USA |  | HM804481 | Family Tree DNA | Direct Submission |
| 181 | JQ705060 | I4a1 |  |  | JQ705060 | [2] | 22482806 |
| 182 | EF153786 | I4a1 | North Asia | Siberia | EF153786 | [19] | 17924343 |
| 183 | Tor807 | I4a | Europe West | Italy, Marche | KF146260 | This Study |  |
| 184 | JQ245737 | I4a | Caucasus North | Ossetia | JQ245737 | [3] | 22284828 |
| 185 | JQ706017 | I4a |  |  | JQ706017 | [2] | 22482806 |
| 186 | JQ701909 | I4a |  |  | JQ701909 | [2] | 22482806 |
| 187 | JQ701957 | I4a |  |  | JQ701957 | [2] | 22482806 |
| 188 | JQ705303 | I4a |  |  | JQ705303 | [2] | 22482806 |
| 189 | EU091245 | I4a | Unknown | - | EU091245 | Family Tree DNA | Direct Submission |
| 190 | HG00154 | I4a | Europe North | Great Britain |  | 1000 Genomes Project |  |
| 191 | Tor802 | I4b | Near East | Iran, Mazandaran | KF146261 | This Study |  |
| 192 | JQ704976 | I4b |  |  | JQ704976 | [2] | 22482806 |
| 193 | EF153785 | N1a1b1 | North Asia | Siberia | EF153785 | [19] | 17924343 |
| 194 | Tor817 | N1a1b1a | Near East | Iran, Yazd | KF146262 | This Study |  |
| 195 | JQ245735 | N1a1b1a1 | Near East | Dubai | JQ245735 | [3] | 22284828 |
| 196 | JQ245734 | N1a1b1a1 | Near East | Dubai | JQ245734 | [3] | 22284828 |

**Table S2.** Origin and sub-haplogroup affiliation of haplogroup W mitogenomes considered in this study.

| Sample # | Sequence ID^a^ | Haplogroup | Region | Country/Ethnicity | GenBank ID | Ref (provided by) | PubMed ID |
| --- | --- | --- | --- | --- | --- | --- | --- |
| 1 | JQ702793 | W1 |  |  | JQ702793 | [2] | 22482806 |
| 2 | JQ704787 | W1a |  |  | JQ704787 | [2] | 22482806 |
| 3 | AY339475 | W1a | Europe North | Finland | AY339475 | [1] | 11349229 |
| 4 | AY339476 | W1a | Europe North | Finland | AY339476 | [1] | 11349229 |
| 5 | AY339477 | W1a | Europe North | Finland | AY339477 | [1] | 11349229 |
| 6 | AY339479 | W1a | Europe North | Finland | AY339479 | [1] | 11349229 |
| 7 | AY339480 | W1a | Europe North | Finland | AY339480 | [1] | 11349229 |
| 8 | AY339483 | W1a | Europe North | Finland | AY339483 | [1] | 11349229 |
| 9 | AY339484 | W1a | Europe North | Finland | AY339484 | [11] | 11553319 |
| 10 | AY339485 | W1a | Europe North | Finland | AY339485 | [1] | 11349229 |
| 11 | AY339486 | W1a | Europe North | Finland | AY339486 | [1] | 11349229 |
| 12 | AY339487 | W1a | Europe North | Finland | AY339487 | [1] | 11349229 |
| 13 | AY339488 | W1a | Europe North | Finland | AY339488 | [1] | 11349229 |
| 14 | HG00308 | W1a | Europe North | Finland |  | 1000 Genomes Project |  |
| 15 | AY339494 | W1a | Europe North | Finland | AY339494 | [1] | 11349229 |
| 16 | AY339493 | W1a | Europe North | Finland | AY339493 | [1] | 11349229 |
| 17 | AY339496 | W1a | Europe North | Finland | AY339496 | [1] | 11349229 |
| 18 | AY339495 | W1a | Europe North | Finland | AY339495 | [1] | 11349229 |
| 19 | AY339491 | W1a | Europe North | Finland | AY339491 | [1] | 11349229 |
| 20 | AY339478 | W1a | Europe North | Finland | AY339478 | [1] | 11349229 |
| 21 | AY339481 | W1a | Europe North | Finland | AY339481 | [1] | 11349229 |
| 22 | AY339482 | W1a | Europe North | Finland | AY339482 | [1] | 11349229 |
| 23 | AY339492 | W1a | Europe North | Finland | AY339492 | [1] | 11349229 |
| 24 | AY195779 | W1a | Europe North | Finland | AY195779 | [9] | 12509511 |
| 25 | AY339490 | W1a | Europe North | Finland | AY339490 | [1] | 11349229 |
| 26 | AY339489 | W1a | Europe North | Finland | AY339489 | [1] | 11349229 |
| 27 | AY339462 | W1b | Europe North | Finland | AY339462 | [1] | 11349229 |
| 28 | AY339464 | W1b | Europe North | Finland | AY339464 | [1] | 11349229 |
| 29 | AY339471 | W1b1 | Europe North | Finland | AY339471 | [1] | 11349229 |
| 30 | AY339466 | W1b1 | Europe North | Finland | AY339466 | [1] | 11349229 |
| 31 | AY339467 | W1b1 | Europe North | Finland | AY339467 | [1] | 11349229 |
| 32 | AY339468 | W1b1 | Europe North | Finland | AY339468 | [1] | 11349229 |
| 33 | AY339469 | W1b1 | Europe North | Finland | AY339469 | [1] | 11349229 |
| 34 | AY339470 | W1b1 | Europe North | Finland | AY339470 | [1] | 11349229 |
| 35 | AY339472 | W1b1 | Europe North | Finland | AY339472 | [1] | 11349229 |
| 36 | JQ702545 | W1b1 |  |  | JQ702545 | [2] | 22482806 |
| 37 | GU123029 | W1b1 | Europe East | Russia | GU123029 | [5] | 20457583 |
| 38 | AY339474 | W1b1 | Europe North | Finland | AY339474 | [1] | 11349229 |
| 39 | AY339473 | W1b1 | Europe North | Finland | AY339473 | [1] | 11349229 |
| 40 | AY339465 | W1b | Europe North | Finland | AY339465 | [1] | 11349229 |
| 41 | AY339463 | W1b | Europe North | Finland | AY339463 | [1] | 11349229 |
| 42 | JQ705839 | W1e |  |  | JQ705839 | [2] | 22482806 |
| 43 | FJ543390 | W1e | Europe North | Finland | FJ543390 | Family Tree DNA | Direct Submission |
| 44 | JQ702638 | W1e |  |  | JQ702638 | [2] | 22482806 |
| 45 | JQ703406 | W1e |  |  | JQ703406 | [2] | 22482806 |
| 46 | Tor771 | W1e | Europe West | Italy, Marche | KF146263 | This Study |  |
| 47 | FJ384431 | W1f | Unknown | - | FJ384431 | [20] | 19331681 |
| 48 | FJ384432 | W1f | Unknown | - | FJ384432 | [20] | 19331681 |
| 49 | FJ348225 | W1f | Europe East | USA, Hutterite from Russia | FJ348225 | [14] | 19844259 |
| 50 | Tor768 | W1c | Europe West | Italy, Umbria | KF146264 | This Study |  |
| 51 | JQ701837 | W1c |  |  | JQ701837 | [2] | 22482806 |
| 52 | JQ704790 | W1c1 |  |  | JQ704790 | [2] | 22482806 |
| 53 | NA12400 | W1c1 | Europe |  |  | 1000 Genomes Project |  |
| 54 | JQ702558 | W1c1 |  |  | JQ702558 | [2] | 22482806 |
| 55 | JQ704828 | W1c1 |  |  | JQ704828 | [2] | 22482806 |
| 56 | JQ702327 | W1c1 |  |  | JQ702327 | [2] | 22482806 |
| 57 | NA07056 | W1c1 | Europe |  |  | 1000 Genomes Project |  |
| 58 | JQ245755 | W1c | Caucasus North | Kabardia | JQ245755 | [3] | 22284828 |
| 59 | JQ706021 | W1c |  |  | JQ706021 | [2] | 22482806 |
| 60 | JQ245768 | W1c | Near East | Turkey | JQ245768 | [3] | 22284828 |
| 61 | Tor781 | W1c | Near East | Iran, Khorasan | KF146265 | This Study |  |
| 62 | AY714039 | W1c | Asia South | India | AY714039 | [8] | 15467980 |
| 63 | EU257638 | W1c | Unknown | - | EU257638 | Family Tree DNA | Direct Submission |
| 64 | HM560726 | W1c | Asia South | India | HM560726 | Family Tree DNA | Direct Submission |
| 65 | JQ702450 | W1c |  |  | JQ702450 | [2] | 22482806 |
| 66 | JQ705036 | W1 |  |  | JQ705036 | [2] | 22482806 |
| 67 | JQ703091 | W1 |  |  | JQ703091 | [2] | 22482806 |
| 68 | Tor792 | W1 | Europe East | Poland | KF146266 | This Study |  |
| 69 | JQ702050 | W1 |  |  | JQ702050 | [2] | 22482806 |
| 70 | JQ705056 | W1 |  |  | JQ705056 | [2] | 22482806 |
| 71 | JQ703013 | W1 |  |  | JQ703013 | [2] | 22482806 |
| 72 | FJ472633 | W1 | Unknown | - | FJ472633 | Family Tree DNA | Direct Submission |
| 73 | EU558696 | W1 | Europe North | England | EU558696 | Family Tree DNA | Direct Submission |
| 74 | GU123002 | W1 | Europe East | Russia | GU123002 | [5] | 20457583 |
| 75 | Tor18 | W1 | Caucasus North | Adygei | KF146267 | This Study |  |
| 76 | JF812162 | W1 | Unknown | - | JF812162 | Family Tree DNA | Direct Submission |
| 77 | JQ703242 | W1 |  |  | JQ703242 | [2] | 22482806 |
| 78 | AY339461 | W1 | Europe North | Finland | AY339461 | [1] | 11349229 |
| 79 | AY339460 | W1 | Europe North | Finland | AY339460 | [1] | 11349229 |
| 80 | EF556154 | W1d | Near East | Iraq | EF556154 | [7] | 18446216 |
| 81 | EF609013 | W1 | Europe East | Poland | EF609013 | Family Tree DNA | Direct Submission |
| 82 | Tor767 | W1 | Near East | Iran, Azerbaijan | KF146268 | This Study |  |
| 83 | JQ704975 | W1g |  |  | JQ704975 | [2] | 22482806 |
| 84 | JQ704989 | W1g |  |  | JQ704989 | [2] | 22482806 |
| 85 | JQ702249 | W1g |  |  | JQ702249 | [2] | 22482806 |
| 86 | Tor785 | W1 | Europe West | Italy, Sardinia | KF146269 | This Study |  |
| 87 | JQ702332 | W1 |  |  | JQ702332 | [2] | 22482806 |
| 88 | Tor788 | W1 | Europe East | Ukraine | KF146270 | This Study |  |
| 89 | JQ703970 | W1 |  |  | JQ703970 | [2] | 22482806 |
| 90 | Tor774 | W1 | Europe West | Italy, Tuscany | KF146271 | This Study |  |
| 91 | HQ593809 | W1 | Europe West | Italy | HQ593809 | [21] | 20978534 |
| 92 | Tor793 | W1 | Europe West | Italy, Apulia | KF146272 | This Study |  |
| 93 | AF382003 | W1 | Europe West | Spain, Maragato | AF382003 | [11] | 11553319 |
| 94 | Tor782 | W3a1 | Europe West | Italy, Tuscany | KF146273 | This Study |  |
| 95 | FJ821289 | W3a1a | Europe North | England | FJ821289 | Family Tree DNA | Direct Submission |
| 96 | JQ705642 | W3a1a2 |  |  | JQ705642 | [2] | 22482806 |
| 97 | GU123000 | W3a1a2 | Europe East | Russia | GU123000 | [5] | 20457583 |
| 98 | JQ705286 | W3a1a1 |  |  | JQ705286 | [2] | 22482806 |
| 99 | FJ472839 | W3a1a1 | Europe East | Poland | FJ472839 | Family Tree DNA | Direct Submission |
| 100 | Tor794 | W3a1 | Europe West | Italy, Sardinia | KF146274 | This Study |  |
| 101 | Tor783 | W3a1 | Europe West | Italy, Campania | KF146275 | This Study |  |
| 102 | AY195768 | W3a1 | Europe | Europe | AY195768 | [9] | 12509511 |
| 103 | JQ704996 | W3a1 |  |  | JQ704996 | [2] | 22482806 |
| 104 | Tor787 | W3a1 | Europe West | France, Toulose | KF146276 | This Study |  |
| 105 | Tor769 | W3a1 | Near East | Morocco, Berber | KF146277 | This Study |  |
| 106 | Tor777 | W3a1b | Asia South | India, New-Delhi | KF146278 | This Study |  |
| 107 | GU002155 | W3a1b | Asia South | India, Punjab | GU002155 | Family Tree DNA | Direct Submission |
| 108 | AY714043 | W3a1b | Asia South | India | AY714043 | [8] | 15467980 |
| 109 | HM156696 | W3a1b | Asia South | India | HM156696 | [10] | 21296687 |
| 110 | HM156692 | W3a1b | Asia South | India | HM156692 | [10] | 21296687 |
| 111 | JQ701844 | W3a1 |  |  | JQ701844 | [2] | 22482806 |
| 112 | JF829690 | W3a1 | Unknown | - | JF829690 | Family Tree DNA | Direct Submission |
| 113 | NA12775 | W3a1 |  |  |  | 1000 Genomes Project |  |
| 114 | JQ702421 | W3a1 |  |  | JQ702421 | [2] | 22482806 |
| 115 | HM156689 | W3a1 | Asia South | India | HM156689 | [10] | 21296687 |
| 116 | JQ245760 | W3a1 | Near East | Turkey | JQ245760 | [3] | 22284828 |
| 117 | GU122989 | W3a1 | Europe East | Russia | GU122989 | [5] | 20457583 |
| 118 | JQ703058 | W3a1c |  |  | JQ703058 | [2] | 22482806 |
| 119 | JQ706024 | W3a1c |  |  | JQ706024 | [2] | 22482806 |
| 120 | JQ702804 | W3a1c |  |  | JQ702804 | [2] | 22482806 |
| 121 | JQ704160 | W3a1 |  |  | JQ704160 | [2] | 22482806 |
| 122 | DQ372887 | W3a1 | Europe | New Zeland, European ancestry | DQ372887 | [22] | 16855009 |
| 123 | JQ705313 | W3a |  |  | JQ705313 | [2] | 22482806 |
| 124 | JQ705487 | W3a2 |  |  | JQ705487 | [2] | 22482806 |
| 125 | GU147938 | W3a2 | Europe North | USA, North European ancestry | GU147938 | Family Tree DNA | Direct Submission |
| 126 | HM214761 | W3a2 | Europe North | Canada, North European ancestry | HM214761 | Family Tree DNA | Direct Submission |
| 127 | JQ245741 | W3 | Caucasus South | Azerbaijan | JQ245741 | [3] | 22284828 |
| 128 | JQ245757 | W3b | Near East | Turkey | JQ245757 | [3] | 22284828 |
| 129 | Tor790 | W3b | Near East | Iran, Kordestan | KF146279 | This Study |  |
| 130 | JQ705605 | W3b |  |  | JQ705605 | [2] | 22482806 |
| 131 | JQ705990 | W3b |  |  | JQ705990 | [2] | 22482806 |
| 132 | JQ615950 | W3b | Canada | Canada | JQ615950 | Family Tree DNA | Direct Submission |
| 133 | FJ461348 | W3b | Europe East | Hungary | FJ461348 | Family Tree DNA | Direct Submission |
| 134 | HM589047 | W3b | Asia South | India | HM589047 | Family Tree DNA | Direct Submission |
| 135 | JQ245743 | W3b | Europe East | Bulgaria | JQ245743 | [3] | 22284828 |
| 136 | JQ705765 | W3b1 |  |  | JQ705765 | [2] | 22482806 |
| 137 | JQ701824 | W3b1 |  |  | JQ701824 | [2] | 22482806 |
| 138 | JQ702298 | W3b1 |  |  | JQ702298 | [2] | 22482806 |
| 139 | Tor789 | W4a | East Asia | Mongolia, Khentii | KF146280 | This Study |  |
| 140 | Tor784 | W4a | Europe East | Poland | KF146281 | This Study |  |
| 141 | AY714018 | W4a | Asia South | India | AY714018 | [8] | 15467980 |
| 142 | HM034304 | W4a | Europe North | England | HM034304 | Family Tree DNA | Direct Submission |
| 143 | EU400619 | W4a | Unknown | - | EU400619 | Family Tree DNA | Direct Submission |
| 144 | JQ703511 | W4a |  |  | JQ703511 | [2] | 22482806 |
| 145 | Tor795 | W4a | Europe West | Italy, Piedmont | KF146282 | This Study |  |
| 146 | GU045487 | W4a | Europe North | Ireland, Ulter Scots | GU045487 | Family Tree DNA | Direct Submission |
| 147 | Tor780 | W4 | Near East | Iran, Lorestan | KF146283 | This Study |  |
| 148 | FJ348217 | W4 | Europe East | USA, Hutterite from Russia | FJ348217 | [14] | 19844259 |
| 149 | FJ348223 | W4 | Europe East | USA, Hutterite from Russia | FJ348223 | [14] | 19844259 |
| 150 | JQ245758 | W4 | Near East | Turkey | JQ245758 | [3] | 22284828 |
| 151 | EF660966 | W4 | Europe West | Italy | EF660966 | [12] | 17517629 |
| 152 | NA20503 | W4 | Europe West | Italy, Tuscany |  | 1000 Genomes Project |  |
| 153 | JF431251 | W5a1a1 | Europe North | England | JF431251 | Family Tree DNA | Direct Submission |
| 154 | HM057816 | W5a1a1a | USA |  | HM057816 | Family Tree DNA | Direct Submission |
| 155 | GU828018 | W5a1a1a | Europe North | England | GU828018 | Family Tree DNA | Direct Submission |
| 156 | HM125971 | W5a1a1 | Europe North | Germany | HM125971 | Family Tree DNA | Direct Submission |
| 157 | EU135972 | W5a1a1 | Unknown | - | EU135972 | Family Tree DNA | Direct Submission |
| 158 | JQ705536 | W5a1a |  |  | JQ705536 | [2] | 22482806 |
| 159 | JQ702098 | W5a1a |  |  | JQ702098 | [2] | 22482806 |
| 160 | NA20762 | W5a1a | Europe West | Italy, Tuscany |  | 1000 Genomes Project |  |
| 161 | HG00231 | W5a1a | Europe North | Great Britain |  | 1000 Genomes Project |  |
| 162 | NA12413 | W5a1a | Europe |  |  | 1000 Genomes Project |  |
| 163 | EF652811 | W5a1a | Europe North | England | EF652811 | Family Tree DNA | Direct Submission |
| 164 | JQ702749 | W5a1a |  |  | JQ702749 | [2] | 22482806 |
| 165 | JQ702061 | W5a1a |  |  | JQ702061 | [2] | 22482806 |
| 166 | GU646872 | W5a1a | Europe North | Ireland, County Offaly | GU646872 | Family Tree DNA | Direct Submission |
| 167 | JF436973 | W5a1a | Europe North | Belgium, Flemish | JF436973 | Family Tree DNA | Direct Submission |
| 168 | Tor772 | W5a1a | Europe West | Italy, Tuscany | KF146284 | This Study |  |
| 169 | JN583886 | W5a1 | Europe North | Ireland, Kilkenny | JN583886 | Family Tree DNA | Direct Submission |
| 170 | NA11933 | W5a1 | Europe |  |  | 1000 Genomes Project |  |
| 171 | HG00148 | W5a2 | Europe North | Great Britain |  | 1000 Genomes Project |  |
| 172 | GU726895 | W5a2 | USA |  | GU726895 | Family Tree DNA | Direct Submission |
| 173 | JQ705278 | W5a2 |  |  | JQ705278 | [2] | 22482806 |
| 174 | JF419335 | W5a2 | Europe North | Ireland, County Antrim | JF419335 | Family Tree DNA | Direct Submission |
| 175 | GU817015 | W5a2 | Europe North | Germany | GU817015 | Family Tree DNA | Direct Submission |
| 176 | JQ701827 | W5a |  |  | JQ701827 | [2] | 22482806 |
| 177 | Tor770 | W5 | Near East | Morocco, Berber | KF146285 | This Study |  |
| 178 | JQ701912 | W5b |  |  | JQ701912 | [2] | 22482806 |
| 179 | JQ703076 | W5b |  |  | JQ703076 | [2] | 22482806 |
| 180 | JQ703283 | W5b |  |  | JQ703283 | [2] | 22482806 |
| 181 | EU744586 | W5b | Unknown | - | EU744586 | Family Tree DNA | Direct Submission |
| 182 | JF902025 | W6 | Europe North | England | JF902025 | Family Tree DNA | Direct Submission |
| 183 | JQ245769 | W6 | Near East | Turkey | JQ245769 | [3] | 22284828 |
| 184 | JQ245736 | W6 | Caucasus North | North Ossetia | JQ245736 | [3] | 22284828 |
| 185 | JQ245723 | W6 | Caucasus North | North Ossetia | JQ245723 | [3] | 22284828 |
| 186 | JQ705793 | W6 |  |  | JQ705793 | [2] | 22482806 |
| 187 | Tor791 | W6 | Europe West | Italy, Umbria | KF146286 | This Study |  |
| 188 | Tor779 | W6 | Near East | Kuwait | KF146287 | This Study |  |
| 189 | FJ449703 | W6 | Unknown | - | FJ449703 | Family Tree DNA | Direct Submission |
| 190 | NA20804 | W6a | Europe West | Italy, Tuscany |  | 1000 Genomes Project |  |
| 191 | JQ705209 | W6a |  |  | JQ705209 | [2] | 22482806 |
| 192 | JQ703084 | W6a |  |  | JQ703084 | [2] | 22482806 |
| 193 | HG00336 | W6a | Europe North | Finland |  | 1000 Genomes Project |  |
| 194 | GQ149695 | W6a | Unknown | - | GQ149695 | Family Tree DNA | Direct Submission |
| 195 | Tor796 | W6 | Europe West | Italy, Piedmont | KF146288 | This Study |  |
| 196 | JQ245762 | W6 | Near East | Israel | JQ245762 | [3] | 22284828 |
| 197 | JF286634 | W6 | Caucasus South | Armenia | JF286634 | Family Tree DNA | Direct Submission |
| 198 | HQ423397 | W6 | Europe East | Poland | HQ423397 | Family Tree DNA | Direct Submission |
| 199 | HM156681 | W6 | Asia South | India | HM156681 | [10] | 21296687 |
| 200 | FJ348172 | W6 | Europe East | USA, Hutterite from Russia | FJ348172 | [14] | 19844259 |
| 201 | Tor773 | W6 | Europe West | Italy, Tuscany | KF146289 | This Study |  |
| 202 | NA20510 | W6 | Europe West | Italy, Tuscany |  | 1000 Genomes Project |  |
| 203 | JQ705108 | W6b |  |  | JQ705108 | [2] | 22482806 |
| 204 | JQ245728 | W6b | Near East | Dubai | JQ245728 | [3] | 22284828 |
| 205 | Tor786 | W6b | Near East | Iran, Fars | KF146290 | This Study |  |
| 206 | FJ473381 | W6b | Unknown | - | FJ473381 | Family Tree DNA | Direct Submission |
| 207 | JQ703497 | W6b |  |  | JQ703497 | [2] | 22482806 |
| 208 | JQ245761 | W6b | Near East | Israel | JQ245761 | [3] | 22284828 |
| 209 | JF275845 | W6c | USA |  | JF275845 | Family Tree DNA | Direct Submission |
| 210 | EU515252 | W6c | Caucasus South | Armenia | EU515252 | Family Tree DNA | Direct Submission |
| 211 | Tor778 | W | Near East | Iran, Khuzistan | KF146291 | This Study |  |
| 212 | JQ702358 | W |  |  | JQ702358 | [2] | 22482806 |
| 213 | JQ245759 | W | Near East | Turkey | JQ245759 | [3] | 22284828 |
| 214 | JQ245778 | W | Near East | Yemen | JQ245778 | [3] | 22284828 |
| 215 | NA19776 | W | USA | USA, Mexican origin |  | 1000 Genomes Project |  |
| 216 | HM352797 | W7 | Unknown | - | HM352797 | Family Tree DNA | Direct Submission |
| 217 | JQ705720 | W7 |  |  | JQ705720 | [2] | 22482806 |
| 218 | JQ702147 | W7 |  |  | JQ702147 | [2] | 22482806 |
| 219 | HQ844617 | W7 | Caucasus South | Armenia | HQ844617 | Family Tree DNA | Direct Submission |
| 220 | Tor776 | W | Near East | Iran, Gilan | KF146292 | This Study |  |
| 221 | JQ702989 | W |  |  | JQ702989 | [2] | 22482806 |
| 222 | EU086510 | W | Unknown | - | EU086510 | Family Tree DNA | Direct Submission |
| 223 | Tor775 | W | Europe West | Italy, Umbria | KF146293 | This Study |  |

**Table S3.** Percentage frequency distribution of haplogroups I and W and the sub-clades I1a, and W6*.*

| Geographic Area |  | Country/Population | I | I1a | W | W6 | N | References |
| --- | --- | --- | --- | --- | --- | --- | --- | --- |
| Asia |  |  |  |  |  |  |  |  |
|  | China |  | 0 | 0 | 0.42 | 0 | 1439 |  |
|  |  | China East | 0 | 0 | 0 | 0 | 221 | [23,24] |
|  |  | China West | 0 | 0 | 1.75 | 0 | 228 | [24-27] |
|  |  | Chiana South | 0 | 0 | 0.20 | 0 | 990 | [24,26,28-31] |
|  | India |  | 0.34 | 0 | 1.39 | 0.19 | 2671 |  |
|  |  | India North (West and Central) | 0.63 | 0 | 6.05 | 0.63 | 479 | [32-36] |
|  |  | India North East | 0.48 | 0 | 0.16 | 0 | 624 | [32-34,36-38] |
|  |  | India Centre | 0.76 | 0 | 0.76 | 0 | 131 | [33,34,38] |
|  |  | India South East | 0.20 | 0 | 0.60 | 0.20 | 1006 | [32-34,36,38,39] |
|  |  | India South West | 0 | 0 | 0 | 0 | 431 | [32,34,40] |
|  |  | Kazakhstan | 0.97 | 0 | 0.97 | 0 | 309 | [25,27,41] |
|  |  | Kyrgystan | 1.21 | 0 | 1.61 | 0.81 | 248 | [41] |
|  |  | Mongolia | 0 | 0 | 0 | 0 | 199 | [19,25,42] |
|  |  | Nepal | 0 | 0 | 0 | 0 | 168 | [36] |
|  | Siberia |  | 0.56 | 0.07 | 0.07 | 0 | 1436 |  |
|  |  | Siberia Eastern | 0 | 0 | 0.22 | 0 | 449 | [19,43-46] |
|  |  | Siberia Centre | 0.59 | 0 | 0 | 0 | 674 | [19] |
|  |  | Siberia Western | 1.28 | 0.32 | 0 | 0 | 313 | [19,44] |
|  |  | Tajikistan | 0.60 | 0 | 5.44 | 0 | 331 | [19,35,41] |
|  |  | Turkmenistan | 1.55 | 0.31 | 1.55 | 0.93 | 322 | [35,41] |
|  |  | Uzbekistan | 2.10 | 0.47 | 2.10 | 0.23 | 429 | [25,35,41] |
| Near East |  |  |  |  |  |  |  |  |
|  |  | Afghanistan | 0 | 0 | 0 | 0 | 98 | [41] |
|  |  | Druze (Lebanon and Israel) | 3.37 | 0 | 1.12 | 0 | 356 | [47,48] |
|  | Iran |  | 2.08 | 0.21 | 2.41 | 0.68 | 2361 |  |
|  |  | Iran North West | 2.30 | 0.22 | 3.50 | 0.55 | 913 | [19,34-36] |
|  |  | Iran North East | 1.46 | 0.29 | 2.34 | 0.88 | 342 | [19,34-36] |
|  |  | Iran Centre | 2.08 | 0.30 | 1.79 | 0.74 | 672 | [19,34-36] |
|  |  | Iran South | 2.07 | 0 | 1.15 | 0.69 | 434 | [19,34-36] |
|  |  | Iraq | 0.38 | 0 | 2.68 | 0.77 | 261 | [36,49,50] |
|  |  | Israel (Palestinians) | 0 | 0 | 2.56 | 2.56 | 117 | [36,49] |
|  |  | Jordan | 2.06 | 1.03 | 0.69 | 0 | 291 | [49,51,52] |
|  |  | Kurds (Turkey) | 3.66 | 1.22 | 6.10 | 0 | 82 | [49,53] |
|  |  | Kuwait | 1.10 | 0 | 1.29 | 0.37 | 544 | [36,54] |
|  |  | Pakistan | 1.13 | 0 | 1.81 | 0.68 | 441 | [33,35] |
|  |  | Saudi Arabia | 0.86 | 0 | 1.03 | 0.52 | 582 | [55,56] |
|  |  | Syria | 0 | 0 | 2.54 | 0 | 118 | [49,57] |
|  |  | Turkey | 2.35 | 0.59 | 2.94 | 0.29 | 340 | [35,49,58] |
|  |  | United Arab Emirates | 3.61 | 0 | 2.81 | 2.01 | 249 | [59] |
|  |  | Yemen | 0.44 | 0 | 0.88 | 0 | 228 | [49,56,60] |
| Caucasus |  |  |  |  |  |  |  |  |
|  |  | Armenia | 1.57 | 0 | 1.05 | 0.52 | 191 | [49] |
|  |  | Azerbaijan | 2.08 | 0 | 4.17 | 0 | 48 | [49] |
|  |  | Caucasus north/Chechnya/Ossetia/Kabardia/Kalmyk Republic | 1.40 | 0 | 1.40 | 0.84 | 356 | [19,35,49] |
|  |  | Georgia | 0.88 | 0 | 6.19 | 5.31 | 113 | [35,53,61] |
| Europe |  |  |  |  |  |  |  |  |
|  |  | Albania | 2.38 | 2.38 | 0 | 0 | 42 | [49] |
|  |  | Austria | 0.80 | 0 | 1.34 | 0.27 | 374 | [62,63] |
|  |  | Balearic Islands | 1.17 | 0 | 0.78 | 0 | 256 | [64,65] |
|  |  | Basque Country | 1.09 | 0 | 0.22 | 0.22 | 457 | [49,66-71] |
|  |  | Belgium | 0 | 0 | 0 | 0 | 50 | [72] |
|  |  | Bosnia-Herzegovina | 2.78 | 0.69 | 1.39 | 0 | 144 | [73] |
|  |  | Bulgary | 1.41 | 0 | 2.51 | 0.20 | 996 | [36,49,74] |
|  |  | Croatia | 3.13 | 0 | 4.17 | 0 | 96 | [75] |
|  |  | Czech Rep. | 3.61 | 2.41 | 1.20 | 0 | 83 | [49] |
|  |  | Denmark | 2.46 | 0.41 | 0.82 | 0 | 244 | [49,76] |
|  |  | England | 2.90 | 0.87 | 0.87 | 0 | 345 | [49,77] |
|  |  | Estonia | 0.67 | 0.67 | 3.36 | 1.34 | 149 | [49] |
|  |  | Finland | 3.46 | 1.23 | 3.95 | 0 | 405 | [36,49,78,79] |
|  |  | France-North | 2.33 | 1.33 | 2.67 | 0 | 600 | [49,70,80] |
|  |  | France-Centre | 1.12 | 0.28 | 1.12 | 0.28 | 358 | [36,49,70,81] |
|  |  | France-South | 2.50 | 0 | 1.67 | 0 | 240 | [70,80] |
|  |  | Germany-North | 2.32 | 0.61 | 2.56 | 0.37 | 819 | [82-87] |
|  |  | Germany-South | 2.93 | 0.53 | 1.33 | 0 | 376 | [85,88,89] |
|  |  | Greece | 2.30 | 0.24 | 1.81 | 0.12 | 827 | [36,49,57,90] |
|  |  | Hungary | 1.34 | 0.73 | 4.27 | 0.98 | 820 | [36,91-93] |
|  |  | Iceland | 4.81 | 1.31 | 0.22 | 0.22 | 457 | [49,94] |
|  |  | Ireland | 3.00 | 0.33 | 2.33 | 0 | 300 | [95] |
|  | Italy |  | 1.60 | 0.41 | 1.64 | 0.43 | 4442 |  |
|  |  | Italy centre | 1.52 | 0.17 | 2.08 | 0.43 | 2305 | [36,37,65,75,96-98] |
|  |  | Italy north | 1.78 | 1.04 | 1.11 | 0.30 | 1346 | [36,75,98] |
|  |  | Italy south | 1.52 | 0 | 1.26 | 0.63 | 791 | [36,49,75,99] |
|  |  | Latvia | 1.67 | 1.34 | 2.34 | 0.33 | 299 | [100] |
|  |  | Lithuania | 3.89 | 2.78 | 1.11 | 1.11 | 180 | [101] |
|  |  | Macedonia | 1.50 | 1.00 | 4.00 | 0.50 | 200 | [102] |
|  |  | Norway | 1.91 | 0.16 | 1.75 | 0 | 628 | [49,77,103] |
|  |  | Poland | 1.32 | 0.51 | 3.45 | 0.81 | 986 | [36,49,104,105] |
|  | Portugal |  | 1.56 | 0.92 | 1.74 | 0.28 | 1089 |  |
|  |  | Portugal centre | 0.50 | 0.25 | 1.25 | 0.50 | 399 | [106-108] |
|  |  | Portugal north | 2.43 | 1.08 | 2.70 | 0.27 | 371 | [106-108] |
|  |  | Portugal south | 1.88 | 1.57 | 1.25 | 0 | 319 | [106-108] |
|  |  | Romania | 0 | 0 | 6.52 | 0 | 92 | [49] |
|  |  | Russia (West) | 2.40 | 1.44 | 3.23 | 1.08 | 835 | [41,49,104,105,109,110] |
|  |  | Saami | 0 | 0 | 0.34 | 0 | 294 | [36,78,111-113] |
|  |  | Sardinia | 0.49 | 0.16 | 1.06 | 0 | 1224 | [36,49,65] |
|  |  | Scotland | 5.00 | 1.08 | 0.83 | 0.08 | 1199 | [77] |
|  |  | Slovakia | 2.93 | 1.55 | 2.07 | 0.17 | 581 | [114,115] |
|  |  | Slovenia | 2.15 | 0.43 | 2.58 | 0.43 | 233 | [73,116] |
|  | Spain |  | 1.39 | 0.38 | 1.45 | 0.13 | 1586 |  |
|  |  | Spain centre | 0.52 | 0 | 2.06 | 0 | 194 | [64,117,118] |
|  |  | Spain north | 1.71 | 0.51 | 1.45 | 0.09 | 1170 | [36,69,71,106,119-122] |
|  |  | Spain south | 0.45 | 0 | 0.90 | 0.45 | 222 | [64,65,69,117,118] |
|  |  | Sweden | 2.37 | 0 | 1.18 | 0.30 | 338 | [36,49,113] |
|  |  | Switzerland | 1.32 | 0.44 | 1.75 | 0 | 228 | [123,124] |
|  |  | Ukraine | 2.73 | 1.82 | 0.91 | 0 | 110 | [36,46] |
|  |  | Wales | 3.26 | 0 | 0 | 0 | 92 | [85] |
| Africa |  |  |  |  |  |  |  |  |
|  |  | Algeria | 0 | 0 | 0 | 0 | 125 | [69,118] |
|  |  | Cameroon | 0 | 0 | 0 | 0 | 104 | [36] |
|  |  | Egypt (not Berbers) | 2.08 | 0 | 0.38 | 0 | 529 | [36,125,126] |
|  |  | Egypt (Berbers) | 0 | 0 | 0 | 0 | 78 | [127] |
|  |  | Ethiopia | 0.59 | 0 | 0 | 0 | 169 | [36,128] |
|  |  | Lybia | 0.25 | 0 | 0 | 0 | 398 | [129,130] |
|  |  | Mauritania & Western Sahara | 0 | 0 | 0 | 0 | 111 | [118,131] |
|  |  | Morocco (Berbers) | 0 | 0 | 0.81 | 0 | 247 | [118,127,131] |
|  |  | Morocco (not Berbers) | 0 | 0 | 0 | 0 | 192 | [65,118,131,132] |
|  |  | Niger | 0 | 0 | 0 | 0 | 33 | [133] |
|  |  | Nigeria | 0 | 0 | 0 | 0 | 115 | [133,134] |
|  |  | Senegal | 0 | 0 | 0 | 0 | 240 | [131,135] |
|  |  | Somalia | 0 | 0 | 0 | 0 | 27 | [133] |
|  |  | Tunisia (not Berbers) | 0.90 | 0 | 0 | 0 | 111 | [118,132] |
|  |  | Tunisia (Berbers) | 2.58 | 0.65 | 0 | 0 | 155 | [136] |

**REFERENCES**

1. Finnilä S, Lehtonen MS, Majamaa K (2001) Phylogenetic network for European mtDNA. Am J Hum Genet 68: 1475-1484.

2. Behar DM, van Oven M, Rosset S, Metspalu M, Loogväli EL, et al. (2012) A "Copernican" reassessment of the human mitochondrial DNA tree from its root. Am J Hum Genet 90: 675-684.

3. Fernandes V, Alshamali F, Alves M, Costa MD, Pereira JB, et al. (2012) The Arabian cradle: mitochondrial relicts of the first steps along the southern route out of Africa. Am J Hum Genet 90: 347-355.

4. Costa MD, Cherni L, Fernandes V, Freitas F, Ammar El Gaaied AB, et al. (2009) Data from complete mtDNA sequencing of Tunisian centenarians: testing haplogroup association and the "golden mean" to longevity. Mech Ageing Dev 130: 222-226.

5. Malyarchuk B, Derenko M, Denisova G, Kravtsova O (2010) Mitogenomic diversity in Tatars from the Volga-Ural region of Russia. Mol Biol Evol 27: 2220-2226.

6. Schönberg A, Theunert C, Li M, Stoneking M, Nasidze I (2011) High-throughput sequencing of complete human mtDNA genomes from the Caucasus and West Asia: high diversity and demographic inferences. Eur J Hum Genet 19: 988-994.

7. Behar DM, Metspalu E, Kivisild T, Rosset S, Tzur S, et al. (2008) Counting the founders: the matrilineal genetic ancestry of the Jewish Diaspora. PLoS One 3: e2062.

8. Palanichamy MG, Sun C, Agrawal S, Bandelt HJ, Kong QP, et al. (2004) Phylogeny of mitochondrial DNA macrohaplogroup N in India, based on complete sequencing: implications for the peopling of South Asia. Am J Hum Genet 75: 966-978.

9. Mishmar D, Ruiz-Pesini E, Golik P, Macaulay V, Clark AG, et al. (2003) Natural selection shaped regional mtDNA variation in humans. Proc Natl Acad Sci U S A 100: 171-176.

10. Govindaraj P, Khan NA, Gopalakrishna P, Chandra RV, Vanniarajan A, et al. (2011) Mitochondrial dysfunction and genetic heterogeneity in chronic periodontitis. Mitochondrion 11: 504-512.

11. Maca-Meyer N, González AM, Larruga JM, Flores C, Cabrera VM (2001) Major genomic mitochondrial lineages delineate early human expansions. BMC Genet 2: 13.

12. Gasparre G, Porcelli AM, Bonora E, Pennisi LF, Toller M, et al. (2007) Disruptive mitochondrial DNA mutations in complex I subunits are markers of oncocytic phenotype in thyroid tumors. Proc Natl Acad Sci U S A 104: 9001-9006.

13. Achilli A, Iommarini L, Olivieri A, Pala M, Hooshiar Kashani B, et al. (2012) Rare primary mitochondrial DNA mutations and probable synergistic variants in Leber's hereditary optic neuropathy. PLoS One 7: e42242.

14. Pichler I, Fuchsberger C, Platzer C, Calişkan M, Marroni F, et al. (2010) Drawing the history of the Hutterite population on a genetic landscape: inference from Y-chromosome and mtDNA genotypes. Eur J Hum Genet 18: 463-470.

15. Janssen GM, Neu A, 't Hart LM, van de Sande CM, Antonie Maassen J (2006) Novel mitochondrial DNA length variants and genetic instability in a family with diabetes and deafness. Exp Clin Endocrinol Diabetes 114: 168-174.

16. Pope AM, Carr SM, Smith KN, Marshall HD (2011) Mitogenomic and microsatellite variation in descendants of the founder population of Newfoundland: high genetic diversity in an historically isolated population. Genome 54: 110-119.

17. Bandelt HJ, Achilli A, Kong QP, Salas A, Lutz-Bonengel S, et al. (2005) Low "penetrance" of phylogenetic knowledge in mitochondrial disease studies. Biochem Biophys Res Commun 333: 122-130.

18. Gonder MK, Mortensen HM, Reed FA, de Sousa A, Tishkoff SA (2007) Whole-mtDNA genome sequence analysis of ancient African lineages. Mol Biol Evol 24: 757-768.

19. Derenko M, Malyarchuk B, Grzybowski T, Denisova G, Dambueva I, et al. (2007) Phylogeographic analysis of mitochondrial DNA in northern Asian populations. Am J Hum Genet 81: 1025-1041.

20. Fendt L, Zimmermann B, Daniaux M, Parson W (2009) Sequencing strategy for the whole mitochondrial genome resulting in high quality sequences. BMC Genomics 10: 139.

21. Zaragoza MV, Brandon MC, Diegoli M, Arbustini E, Wallace DC (2011) Mitochondrial cardiomyopathies: how to identify candidate pathogenic mutations by mitochondrial DNA sequencing, MITOMASTER and phylogeny. Eur J Hum Genet 19: 200-207.

22. Pierson MJ, Martinez-Arias R, Holland BR, Gemmell NJ, Hurles ME, et al. (2006) Deciphering past human population movements in Oceania: provably optimal trees of 127 mtDNA genomes. Mol Biol Evol 23: 1966-1975.

23. Nishimaki Y, Sato K, Fang L, Ma M, Hasekura H, et al. (1999) Sequence polymorphism in the mtDNA HV1 region in Japanese and Chinese. Leg Med (Tokyo) 1: 238-249.

24. Yao YG, Kong QP, Bandelt HJ, Kivisild T, Zhang YP (2002) Phylogeographic differentiation of mitochondrial DNA in Han Chinese. Am J Hum Genet 70: 635-651.

25. Yao YG, Kong QP, Wang CY, Zhu CL, Zhang YP (2004) Different matrilineal contributions to genetic structure of ethnic groups in the silk road region in China. Mol Biol Evol 21: 2265-2280.

26. Yao YG, Nie L, Harpending H, Fu YX, Yuan ZG, et al. (2002) Genetic relationship of Chinese ethnic populations revealed by mtDNA sequence diversity. Am J Phys Anthropol 118: 63-76.

27. Yao YG, Lü XM, Luo HR, Li WH, Zhang YP (2000) Gene admixture in the silk road region of China: evidence from mtDNA and melanocortin 1 receptor polymorphism. Genes Genet Syst 75: 173-178.

28. Chen F, Wang SY, Zhang RZ, Hu YH, Gao GF, et al. (2008) Analysis of mitochondrial DNA polymorphisms in Guangdong Han Chinese. Forensic Sci Int Genet 2: 150-153.

29. Irwin JA, Saunier JL, Beh P, Strouss KM, Paintner CD, et al. (2009) Mitochondrial DNA control region variation in a population sample from Hong Kong, China. Forensic Sci Int Genet 3: e119-125.

30. Yao YG, Zhang YP (2002) Phylogeographic analysis of mtDNA variation in four ethnic populations from Yunnan Province: new data and a reappraisal. J Hum Genet 47: 311-318.

31. Kivisild T, Tolk HV, Parik J, Wang Y, Papiha SS, et al. (2002) The emerging limbs and twigs of the East Asian mtDNA tree. Mol Biol Evol 19: 1737-1751.

32. Cordaux R, Saha N, Bentley GR, Aunger R, Sirajuddin SM, et al. (2003) Mitochondrial DNA analysis reveals diverse histories of tribal populations from India. Eur J Hum Genet 11: 253-264.

33. Kivisild T, Bamshad MJ, Kaldma K, Metspalu M, Metspalu E, et al. (1999) Deep common ancestry of Indian and Western-Eurasian mitochondrial DNA lineages. Curr Biol 9: 1331-1334.

34. Metspalu M, Kivisild T, Metspalu E, Parik J, Hudjashov G, et al. (2004) Most of the extant mtDNA boundaries in south and southwest Asia were likely shaped during the initial settlement of Eurasia by anatomically modern humans. BMC Genet 5: 26.

35. Quintana-Murci L, Chaix R, Wells RS, Behar DM, Sayar H, et al. (2004) Where west meets east: the complex mtDNA landscape of the southwest and Central Asian corridor. Am J Hum Genet 74: 827-845.

36. Torroni A, Unpublished Data.

37. Achilli A, Olivieri A, Pala M, Metspalu E, Fornarino S, et al. (2007) Mitochondrial DNA variation of modern Tuscans supports the Near Eastern origin of Etruscans. Am J Hum Genet 80: 759-768.

38. Roychoudhury S, Roy S, Basu A, Banerjee R, Vishwanathan H, et al. (2001) Genomic structures and population histories of linguistically distinct tribal groups of India. Hum Genet 109: 339-350.

39. Bamshad MJ, Watkins WS, Dixon ME, Jorde LB, Rao BB, et al. (1998) Female gene flow stratifies Hindu castes. Nature 395: 651-652.

40. Mountain JL, Hebert JM, Bhattacharyya S, Underhill PA, Ottolenghi C, et al. (1995) Demographic history of India and mtDNA-sequence diversity. Am J Hum Genet 56: 979-992.

41. Irwin JA, Ikramov A, Saunier J, Bodner M, Amory S, et al. (2010) The mtDNA composition of Uzbekistan: a microcosm of Central Asian patterns. Int J Legal Med 124: 195-204.

42. Kolman CJ, Sambuughin N, Bermingham E (1996) Mitochondrial DNA analysis of Mongolian populations and implications for the origin of New World founders. Genetics 142: 1321-1334.

43. Schurr TG, Sukernik RI, Starikovskaya YB, Wallace DC (1999) Mitochondrial DNA variation in Koryaks and Itel'men: population replacement in the Okhotsk Sea-Bering Sea region during the Neolithic. Am J Phys Anthropol 108: 1-39.

44. Shields GF, Schmiechen AM, Frazier BL, Redd A, Voevoda MI, et al. (1993) mtDNA sequences suggest a recent evolutionary divergence for Beringian and northern North American populations. Am J Hum Genet 53: 549-562.

45. Starikovskaya YB, Sukernik RI, Schurr TG, Kogelnik AM, Wallace DC (1998) mtDNA diversity in Chukchi and Siberian Eskimos: implications for the genetic history of Ancient Beringia and the peopling of the New World. Am J Hum Genet 63: 1473-1491.

46. Malyarchuk BA, Derenko MV (2001) Mitochondrial DNA variability in Russians and Ukrainians: implication to the origin of the Eastern Slavs. Ann Hum Genet 65: 63-78.

47. Macaulay V, Richards M, Hickey E, Vega E, Cruciani F, et al. (1999) The emerging tree of West Eurasian mtDNAs: a synthesis of control-region sequences and RFLPs. Am J Hum Genet 64: 232-249.

48. Shlush LI, Behar DM, Yudkovsky G, Templeton A, Hadid Y, et al. (2008) The Druze: a population genetic refugium of the Near East. PLoS One 3: e2105.

49. Richards M, Macaulay V, Hickey E, Vega E, Sykes B, et al. (2000) Tracing European founder lineages in the Near Eastern mtDNA pool. Am J Hum Genet 67: 1251-1276.

50. Al-Zahery N, Pala M, Battaglia V, Grugni V, Hamod MA, et al. (2011) In search of the genetic footprints of Sumerians: a survey of Y-chromosome and mtDNA variation in the Marsh Arabs of Iraq. Bmc Evolutionary Biology 11.

51. González-Andrade F, Sánchez D, Martínez-Jarreta B, Budowle B (2008) Y-chromosome STR haplotypes in three different population groups from Ecuador (South America). J Forensic Sci 53: 512-514.

52. González AM, Karadsheh N, Maca-Meyer N, Flores C, Cabrera VM, et al. (2008) Mitochondrial DNA variation in Jordanians and their genetic relationship to other Middle East populations. Ann Hum Biol 35: 212-231.

53. Comas D, Calafell F, Bendukidze N, Fañanás L, Bertranpetit J (2000) Georgian and kurd mtDNA sequence analysis shows a lack of correlation between languages and female genetic lineages. Am J Phys Anthropol 112: 5-16.

54. Scheible M, Alenizi M, Sturk-Andreaggi K, Coble MD, Ismael S, et al. (2011) Mitochondrial DNA control region variation in a Kuwaiti population sample. Forensic Sci Int Genet 5: e112-113.

55. Abu-Amero KK, Larruga JM, Cabrera VM, González AM (2008) Mitochondrial DNA structure in the Arabian Peninsula. BMC Evol Biol 8: 45.

56. Di Rienzo A, Wilson AC (1991) Branching pattern in the evolutionary tree for human mitochondrial DNA. Proc Natl Acad Sci U S A 88: 1597-1601.

57. Vernesi C, Di Benedetto G, Caramelli D, Secchieri E, Simoni L, et al. (2001) Genetic characterization of the body attributed to the evangelist Luke. Proc Natl Acad Sci U S A 98: 13460-13463.

58. Di Benedetto G, Ergüven A, Stenico M, Castrì L, Bertorelle G, et al. (2001) DNA diversity and population admixture in Anatolia. Am J Phys Anthropol 115: 144-156.

59. Alshamali F, Brandstätter A, Zimmermann B, Parson W (2008) Mitochondrial DNA control region variation in Dubai, United Arab Emirates. Forensic Sci Int Genet 2: e9-10.

60. Cerný V, Mulligan CJ, Rídl J, Zaloudková M, Edens CM, et al. (2008) Regional differences in the distribution of the sub-Saharan, West Eurasian, and South Asian mtDNA lineages in Yemen. Am J Phys Anthropol 136: 128-137.

61. Alfonso-Sánchez MA, Martínez-Bouzas C, Castro A, Peña JA, Fernández-Fernández I, et al. (2006) Sequence polymorphisms of the mtDNA control region in a human isolate: the Georgians from Swanetia. J Hum Genet 51: 429-439.

62. Brandstätter A, Niederstätter H, Pavlic M, Grubwieser P, Parson W (2007) Generating population data for the EMPOP database - an overview of the mtDNA sequencing and data evaluation processes considering 273 Austrian control region sequences as example. Forensic Sci Int 166: 164-175.

63. Parson W, Parsons TJ, Scheithauer R, Holland MM (1998) Population data for 101 Austrian Caucasian mitochondrial DNA d-loop sequences: application of mtDNA sequence analysis to a forensic case. Int J Legal Med 111: 124-132.

64. Picornell A, Gómez-Barbeito L, Tomàs C, Castro JA, Ramon MM (2005) Mitochondrial DNA HVRI variation in Balearic populations. Am J Phys Anthropol 128: 119-130.

65. Falchi A, Giovannoni L, Calo CM, Piras IS, Moral P, et al. (2006) Genetic history of some western Mediterranean human isolates through mtDNA HVR1 polymorphisms. J Hum Genet 51: 9-14.

66. Alfonso-Sánchez MA, Cardoso S, Martínez-Bouzas C, Peña JA, Herrera RJ, et al. (2008) Mitochondrial DNA haplogroup diversity in Basques: a reassessment based on HVI and HVII polymorphisms. Am J Hum Biol 20: 154-164.

67. Bertranpetit J, Sala J, Calafell F, Underhill PA, Moral P, et al. (1995) Human mitochondrial DNA variation and the origin of Basques. Ann Hum Genet 59: 63-81.

68. Afonso Costa H, Carvalho M, Lopes V, Balsa F, Bento AM, et al. (2010) Mitochondrial DNA sequence analysis of a native Bolivian population. J Forensic Leg Med 17: 247-253.

69. Côrte-Real HB, Macaulay VA, Richards MB, Hariti G, Issad MS, et al. (1996) Genetic diversity in the Iberian Peninsula determined from mitochondrial sequence analysis. Ann Hum Genet 60: 331-350.

70. Richard C, Pennarun E, Kivisild T, Tambets K, Tolk HV, et al. (2007) An mtDNA perspective of French genetic variation. Ann Hum Biol 34: 68-79.

71. Cardoso S, Zarrabeitia MT, Valverde L, Odriozola A, Alfonso-Sánchez M, et al. (2010) Variability of the entire mitochondrial DNA control region in a human isolate from the Pas Valley (northern Spain). J Forensic Sci 55: 1196-1201.

72. Decorte R, Jehaes E, Xiao FX, Cassiman JJ (1996) Genetic analysis of single hair shafts by automated sequence analysis of the mitochondrial D-loop region. Advances in Forensic Haemogenetics 6. Carracedo, A., Brinkmann, B., Bärr, W. (eds) ed. Berlin: Springer-Verlag. pp. 17-19.

73. Malyarchuk BA, Grzybowski T, Derenko MV, Czarny J, Drobnic K, et al. (2003) Mitochondrial DNA variability in Bosnians and Slovenians. Ann Hum Genet 67: 412-425.

74. Karachanak S, Carossa V, Nesheva D, Olivieri A, Pala M, et al. (2012) Bulgarians vs the other European populations: a mitochondrial DNA perspective. Int J Legal Med 126: 497-503.

75. Babalini C, Martínez-Labarga C, Tolk HV, Kivisild T, Giampaolo R, et al. (2005) The population history of the Croatian linguistic minority of Molise (southern Italy): a maternal view. Eur J Hum Genet 13: 902-912.

76. Mikkelsen M, Sørensen E, Rasmussen EM, Morling N (2010) Mitochondrial DNA HV1 and HV2 variation in Danes. Forensic Sci Int Genet 4: e87-88.

77. Helgason A, Hickey E, Goodacre S, Bosnes V, Stefánsson K, et al. (2001) mtDna and the islands of the North Atlantic: estimating the proportions of Norse and Gaelic ancestry. Am J Hum Genet 68: 723-737.

78. Lahermo P, Sajantila A, Sistonen P, Lukka M, Aula P, et al. (1996) The genetic relationship between the Finns and the Finnish Saami (Lapps): analysis of nuclear DNA and mtDNA. Am J Hum Genet 58: 1309-1322.

79. Hedman M, Brandstätter A, Pimenoff V, Sistonen P, Palo JU, et al. (2007) Finnish mitochondrial DNA HVS-I and HVS-II population data. Forensic Sci Int 172: 171-178.

80. Dubut V, Chollet L, Murail P, Cartault F, Béraud-Colomb E, et al. (2004) mtDNA polymorphisms in five French groups: importance of regional sampling. Eur J Hum Genet 12: 293-300.

81. Rousselet F, Mangin P (1998) Mitochondrial DNA polymorphisms: a study of 50 French Caucasian individuals and application to forensic casework. Int J Legal Med 111: 292-298.

82. Baasner A, Schäfer C, Junge A, Madea B (1998) Polymorphic sites in human mitochondrial DNA control region sequences: population data and maternal inheritance. Forensic Sci Int 98: 169-178.

83. Hofmann S, Jaksch M, Bezold R, Mertens S, Aholt S, et al. (1997) Population genetics and disease susceptibility: characterization of central European haplogroups by mtDNA gene mutations, correlation with D loop variants and association with disease. Hum Mol Genet 6: 1835-1846.

84. Pfeiffer H, Brinkmann B, Hühne J, Rolf B, Morris AA, et al. (1999) Expanding the forensic German mitochondrial DNA control region database: genetic diversity as a function of sample size and microgeography. Int J Legal Med 112: 291-298.

85. Richards M, Côrte-Real H, Forster P, Macaulay V, Wilkinson-Herbots H, et al. (1996) Paleolithic and neolithic lineages in the European mitochondrial gene pool. Am J Hum Genet 59: 185-203.

86. Tetzlaff S, Brandstätter A, Wegener R, Parson W, Weirich V (2007) Mitochondrial DNA population data of HVS-I and HVS-II sequences from a northeast German sample. Forensic Sci Int 172: 218-224.

87. Poetsch M, Wittig H, Krause D, Lignitz E (2003) Mitochondrial diversity of a northeast German population sample. Forensic Sci Int 137: 125-132.

88. Brandstätter A, Klein R, Duftner N, Wiegand P, Parson W (2006) Application of a quasi-median network analysis for the visualization of character conflicts to a population sample of mitochondrial DNA control region sequences from southern Germany (Ulm). Int J Legal Med 120: 310-314.

89. Lutz S, Weisser HJ, Heizmann J, Pollak S (1998) Location and frequency of polymorphic positions in the mtDNA control region of individuals from Germany. Int J Legal Med 111: 67-77.

90. Irwin J, Saunier J, Strouss K, Paintner C, Diegoli T, et al. (2008) Mitochondrial control region sequences from northern Greece and Greek Cypriots. Int J Legal Med 122: 87-89.

91. Brandstätter A, Egyed B, Zimmermann B, Duftner N, Padar Z, et al. (2007) Migration rates and genetic structure of two Hungarian ethnic groups in Transylvania, Romania. Ann Hum Genet 71: 791-803.

92. Brandstätter A, Egyed B, Zimmermann B, Tordai A, Padar Z, et al. (2008) Mitochondrial DNA control region variation in Ashkenazi Jews from Hungary. Forensic Sci Int Genet 2: e4-6.

93. Irwin J, Egyed B, Saunier J, Szamosi G, O'Callaghan J, et al. (2007) Hungarian mtDNA population databases from Budapest and the Baranya county Roma. Int J Legal Med 121: 377-383.

94. Helgason A, Sigureth ardóttir S, Nicholson J, Sykes B, Hill EW, et al. (2000) Estimating Scandinavian and Gaelic ancestry in the male settlers of Iceland. Am J Hum Genet 67: 697-717.

95. McEvoy B, Richards M, Forster P, Bradley DG (2004) The Longue Durée of genetic ancestry: multiple genetic marker systems and Celtic origins on the Atlantic facade of Europe. Am J Hum Genet 75: 693-702.

96. Achilli A Unpublished data.

97. Francalacci P, Bertranpetit J, Calafell F, Underhill PA (1996) Sequence diversity of the control region of mitochondrial DNA in Tuscany and its implications for the peopling of Europe. Am J Phys Anthropol 100: 443-460.

98. Turchi C, Buscemi L, Previderè C, Grignani P, Brandstätter A, et al. (2008) Italian mitochondrial DNA database: results of a collaborative exercise and proficiency testing. Int J Legal Med 122: 199-204.

99. Vona G, Ghiani ME, Calò CM, Vacca L, Memmì M, et al. (2001) Mitochondrial DNA sequence analysis in Sicily. Am J Hum Biol 13: 576-589.

100. Pliss L, Tambets K, Loogväli EL, Pronina N, Lazdins M, et al. (2006) Mitochondrial DNA portrait of Latvians: towards the understanding of the genetic structure of Baltic-speaking populations. Ann Hum Genet 70: 439-458.

101. Kasperaviciūte D, Kucinskas V, Stoneking M (2004) Y chromosome and mitochondrial DNA variation in Lithuanians. Ann Hum Genet 68: 438-452.

102. Zimmermann B, Brandstätter A, Duftner N, Niederwieser D, Spiroski M, et al. (2007) Mitochondrial DNA control region population data from Macedonia. Forensic Sci Int Genet 1: e4-9.

103. Passarino G, Cavalleri GL, Lin AA, Cavalli-Sforza LL, Børresen-Dale AL, et al. (2002) Different genetic components in the Norwegian population revealed by the analysis of mtDNA and Y chromosome polymorphisms. Eur J Hum Genet 10: 521-529.

104. Malyarchuk BA, Grzybowski T, Derenko MV, Czarny J, Woźniak M, et al. (2002) Mitochondrial DNA variability in Poles and Russians. Ann Hum Genet 66: 261-283.

105. Grzybowski T, Malyarchuk BA, Derenko MV, Perkova MA, Bednarek J, et al. (2007) Complex interactions of the Eastern and Western Slavic populations with other European groups as revealed by mitochondrial DNA analysis. Forensic Sci Int Genet 1: 141-147.

106. González AM, Brehm A, Pérez JA, Maca-Meyer N, Flores C, et al. (2003) Mitochondrial DNA affinities at the Atlantic fringe of Europe. Am J Phys Anthropol 120: 391-404.

107. Pereira L, Prata MJ, Amorim A (2000) Diversity of mtDNA lineages in Portugal: not a genetic edge of European variation. Ann Hum Genet 64: 491-506.

108. Pereira L, Cunha C, Amorim A (2004) Predicting sampling saturation of mtDNA haplotypes: an application to an enlarged Portuguese database. Int J Legal Med 118: 132-136.

109. Malyarchuk B, Grzybowski T, Derenko M, Perkova M, Vanecek T, et al. (2008) Mitochondrial DNA phylogeny in Eastern and Western Slavs. Mol Biol Evol 25: 1651-1658.

110. Orekhov V, Poltoraus A, Zhivotovsky LA, Spitsyn V, Ivanov P, et al. (1999) Mitochondrial DNA sequence diversity in Russians. FEBS Lett 445: 197-201.

111. Delghandi M, Utsi E, Krauss S (1998) Saami mitochondrial DNA reveals deep maternal lineage clusters. Hum Hered 48: 108-114.

112. Sajantila A, Lahermo P, Anttinen T, Lukka M, Sistonen P, et al. (1995) Genes and languages in Europe: an analysis of mitochondrial lineages. Genome Res 5: 42-52.

113. Tillmar AO, Coble MD, Wallerström T, Holmlund G (2010) Homogeneity in mitochondrial DNA control region sequences in Swedish subpopulations. Int J Legal Med 124: 91-98.

114. Malyarchuk BA, Perkova MA, Derenko MV, Vanecek T, Lazur J, et al. (2008) Mitochondrial DNA variability in Slovaks, with application to the Roma origin. Ann Hum Genet 72: 228-240.

115. Lehocký I, Baldovic M, Kádasi L, Metspalu E (2008) A database of mitochondrial DNA hypervariable regions I and II sequences of individuals from Slovakia. Forensic Sci Int Genet 2: e53-59.

116. Zupanic Pajnic I, Balazic J, Komel R (2004) Sequence polymorphism of the mitochondrial DNA control region in the Slovenian population. Int J Legal Med 118: 1-4.

117. Larruga JM, Díez F, Pinto FM, Flores C, González AM (2001) Mitochondrial DNA characterisation of European isolates: the Maragatos from Spain. Eur J Hum Genet 9: 708-716.

118. Plaza S, Calafell F, Helal A, Bouzerna N, Lefranc G, et al. (2003) Joining the pillars of Hercules: mtDNA sequences show multidirectional gene flow in the western Mediterranean. Ann Hum Genet 67: 312-328.

119. Alvarez-Iglesias V, Mosquera-Miguel A, Cerezo M, Quintáns B, Zarrabeitia MT, et al. (2009) New population and phylogenetic features of the internal variation within mitochondrial DNA macro-haplogroup R0. PLoS One 4: e5112.

120. Crespillo M, Luque JA, Paredes M, Fernández R, Ramírez E, et al. (2000) Mitochondrial DNA sequences for 118 individuals from northeastern Spain. Int J Legal Med 114: 130-132.

121. Salas A, Comas D, Lareu MV, Bertranpetit J, Carracedo A (1998) mtDNA analysis of the Galician population: a genetic edge of European variation. Eur J Hum Genet 6: 365-375.

122. Maca-Meyer N, Sánchez-Velasco P, Flores C, Larruga JM, González AM, et al. (2003) Y chromosome and mitochondrial DNA characterization of Pasiegos, a human isolate from Cantabria (Spain). Ann Hum Genet 67: 329-339.

123. Pult I, Sajantila A, Simanainen J, Georgiev O, Schaffner W, et al. (1994) Mitochondrial DNA sequences from Switzerland reveal striking homogeneity of European populations. Biol Chem Hoppe Seyler 375: 837-840.

124. Dimo-Simonin N, Grange F, Taroni F, Brandt-Casadevall C, Mangin P (2000) Forensic evaluation of mtDNA in a population from south west Switzerland. Int J Legal Med 113: 89-97.

125. Stevanovitch A, Gilles A, Bouzaid E, Kefi R, Paris F, et al. (2004) Mitochondrial DNA sequence diversity in a sedentary population from Egypt. Ann Hum Genet 68: 23-39.

126. Saunier JL, Irwin JA, Strouss KM, Ragab H, Sturk KA, et al. (2009) Mitochondrial control region sequences from an Egyptian population sample. Forensic Sci Int Genet 3: e97-103.

127. Coudray C, Olivieri A, Achilli A, Pala M, Melhaoui M, et al. (2009) The complex and diversified mitochondrial gene pool of Berber populations. Ann Hum Genet 73: 196-214.

128. Thomas MG, Weale ME, Jones AL, Richards M, Smith A, et al. (2002) Founding mothers of Jewish communities: geographically separated Jewish groups were independently founded by very few female ancestors. Am J Hum Genet 70: 1411-1420.

129. Fadhlaoui-Zid K, Rodríguez-Botigué L, Naoui N, Benammar-Elgaaied A, Calafell F, et al. (2011) Mitochondrial DNA structure in North Africa reveals a genetic discontinuity in the Nile Valley. Am J Phys Anthropol 145: 107-117.

130. Ottoni C, Martínez-Labarga C, Loogväli EL, Pennarun E, Achilli A, et al. (2009) First genetic insight into Libyan Tuaregs: a maternal perspective. Ann Hum Genet 73: 438-448.

131. Rando JC, Pinto F, González AM, Hernández M, Larruga JM, et al. (1998) Mitochondrial DNA analysis of northwest African populations reveals genetic exchanges with European, near-eastern, and sub-Saharan populations. Ann Hum Genet 62: 531-550.

132. Turchi C, Buscemi L, Giacchino E, Onofri V, Fendt L, et al. (2009) Polymorphisms of mtDNA control region in Tunisian and Moroccan populations: an enrichment of forensic mtDNA databases with Northern Africa data. Forensic Sci Int Genet 3: 166-172.

133. Watson E, Forster P, Richards M, Bandelt HJ (1997) Mitochondrial footprints of human expansions in Africa. Am J Hum Genet 61: 691-704.

134. Vigilant L, Stoneking M, Harpending H, Hawkes K, Wilson AC (1991) African populations and the evolution of human mitochondrial DNA. Science 253: 1503-1507.

135. Graven L, Passarino G, Semino O, Boursot P, Santachiara-Benerecetti S, et al. (1995) Evolutionary correlation between control region sequence and restriction polymorphisms in the mitochondrial genome of a large Senegalese Mandenka sample. Mol Biol Evol 12: 334-345.

136. Fadhlaoui-Zid K, Plaza S, Calafell F, Ben Amor M, Comas D, et al. (2004) Mitochondrial DNA heterogeneity in Tunisian Berbers. Ann Hum Genet 68: 222-233.
